# Reduced expression of OXPHOS and DNA damage genes is linked to protection from microvascular complications in long-term type 1 diabetes: the PROLONG study

**DOI:** 10.1038/s41598-021-00183-z

**Published:** 2021-10-20

**Authors:** Türküler Özgümüş, Oksana Sulaieva, Leon Eyrich Jessen, Ruchi Jain, Henrik Falhammar, Thomas Nyström, Sergiu-Bogdan Catrina, Gun Jörneskog, Leif Groop, Mats Eliasson, Björn Eliasson, Kerstin Brismar, Tomasz Stokowy, Peter M. Nilsson, Valeriya Lyssenko

**Affiliations:** 1 Department of Clinical Science, Center for Diabetes Research, University of Bergen, 5032 Bergen, Norway; 2 Medical Laboratory CSD, Vasylkivska Str. 45, Kyiv, Ukraine; 3 Department of Clinical Sciences/Genomics, Diabetes and Endocrinology, Lund University Diabetes Centre, 205 02 Malmö, Sweden; 4 Section for Bioinformatics, Department of Health Technology, Technical University of Denmark, Lyngby, Denmark; 5 Department of Molecular Medicine and Surgery, Karolinska Institute, Stockholm, Sweden; 6 Department of Endocrinology, Metabolism and Diabetes, Karolinska University Hospital, Stockholm, Sweden; 7 Unit for Diabetes Research, Division of Internal Medicine, Department of Clinical Science and Education, Karolinska Institute, South Hospital, Stockholm, Sweden; 8 Center for Diabetes, Academic Specialist Centrum, Stockholm, Sweden; 9 Division of Internal Medicine, Department of Clinical Sciences, Karolinska Institute, Danderyd University Hospital, Stockholm, Sweden; 10 Institute for Molecular Medicine Finland FIMM, University of Helsinki, Helsinki, Finland; 11 Sunderby Research Unit, Department of Public Health and Clinical Medicine, Umeå University, Umeå, Sweden; 12 Department of Medicine, University of Gothenburg, Gothenburg, Sweden; 13 Department of Clinical Science, University of Bergen, 5021 Bergen, Norway

**Keywords:** Diabetes complications, High-throughput screening

## Abstract

Type 1 diabetes is a chronic autoimmune disease requiring insulin treatment for survival. Prolonged duration of type 1 diabetes is associated with increased risk of microvascular complications. Although chronic hyperglycemia and diabetes duration have been considered as the major risk factors for vascular complications, this is not universally seen among all patients. Persons with long-term type 1 diabetes who have remained largely free from vascular complications constitute an ideal group for investigation of natural defense mechanisms against prolonged exposure of diabetes. Transcriptomic signatures obtained from RNA sequencing of the peripheral blood cells were analyzed in non-progressors with more than 30 years of diabetes duration and compared to the patients who progressed to microvascular complications within a shorter duration of diabetes. Analyses revealed that non-progressors demonstrated a reduction in expression of the oxidative phosphorylation (OXPHOS) genes, which were positively correlated with the expression of DNA repair enzymes, namely genes involved in base excision repair (BER) machinery. Reduced expression of OXPHOS and BER genes was linked to decrease in expression of inflammation-related genes, higher glucose disposal rate and reduced measures of hepatic fatty liver. Results from the present study indicate that at transcriptomic level reduction in OXPHOS, DNA repair and inflammation-related genes is linked to better insulin sensitivity and protection against microvascular complications in persons with long-term type 1 diabetes.

## Introduction

Type 1 diabetes is a life-long disease characterized by the destruction of pancreatic beta cells and lack of insulin production. Type 1 diabetes affects patients of any age, but it is the most prevalent form of diabetes for people younger than 20 years old comprising over 85% of all diabetes cases in this age category worldwide ^1^. With insulin treatment, the life expectancy of the people with type 1 diabetes has become comparably longer in relation to general population, which resulted in elevated burden of chronic life-threatening microvascular and macrovascular complications ^2^. The most prevalent of the former is diabetic retinopathy (DR) and diabetic nephropathy (diabetic kidney disease, DKD). The overall prevalence of DR in persons with diabetes (about 77% with type 1 diabetes) was estimated to be 35% while vision threatening DR was found in 12% ^3^. Similarly, DKD represents the major cause of end-stage renal failure ^4,5^.

DR and DKD cause major disabilities in persons with type 1 diabetes, decreasing their health-related quality of life and promote premature death. The risk of microvascular complications increases with poor glycemic control and duration of type 1 diabetes. Typically, it takes about 15 years for the microvascular complications to become manifest ^6^. However, the risk of microvascular complications broadly differs among people with type 1 diabetes ^7^. Of particular interest are persons who remain largely free from vascular complications despite long duration of the disease ^8^. We and others have previously demonstrated that enhanced insulin sensitivity contribute to freedom from vascular complications, but the underlying molecular mechanisms and their possible transcriptomic regulation remain unknown ^9,10^.

The healthy vasculature is characterized by a high metabolic rate. Mitochondrial dysfunction as well as oxidative stress are important contributors to the development of DR and DKD ^11,12^. It has been proposed that excessive activation of enzymes involved in oxidative phosphorylation (OXPHOS) leads to overproduction of reactive oxygen species (ROS), which, in turn, can contribute to the cell damage and trigger inflammatory response ^13^. Nevertheless, therapeutic targeting of the ROS system has until now failed to reduce the risk of microvascular complications. This, however, does not exclude the ROS system as an important player in the development of complications. We rather need better designed studies to find a convincing answer to this important question.

**Figure 1:**
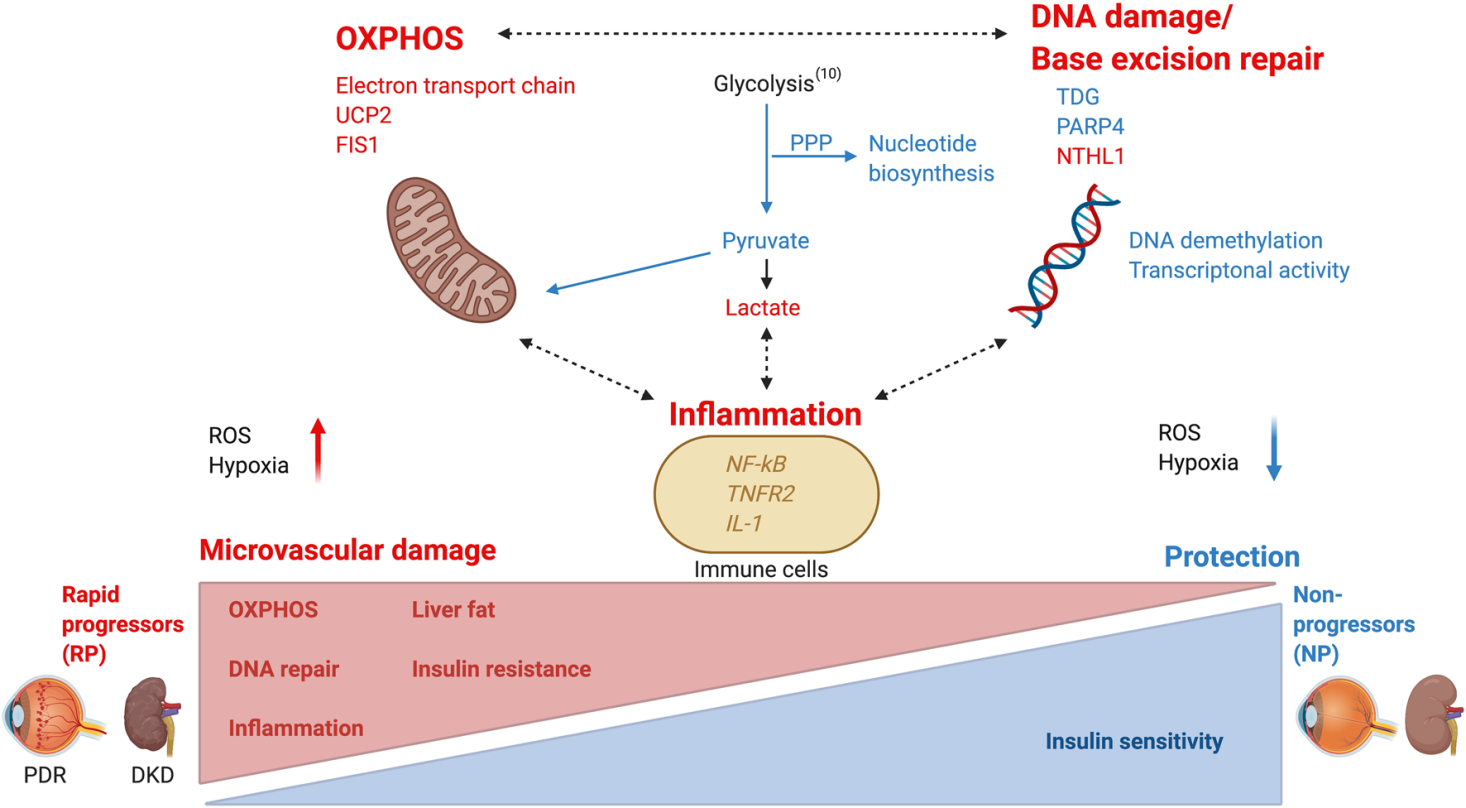
Pathways involved in the progression to microvascular complications (red color) and protective mechanisms (blue color) in patients with prolonged type 1 diabetes. Altered glucose metabolism is associated with a disbalance between glycolysis and oxidative phosphorylation (OXPHOS). A shift towards enhanced OXPHOS in rapid progressors is associated with increased uncoupling between oxidation and phosphorylation, generation of reactive oxygen radicals (ROS) in mitochondria, induction of DNA damage response pathways and pro-inflammatory activation of the immune cells. These mechanisms are involved in progression to microvascular damage and metabolic violations increasing risk of PDR and DKD. On the contrary, non-progressors are characterized by a moderate reduction in expression of OXPHOS genes that correlated with decreased DNA damage and immune cell activation, and linked to better insulin sensitivity and lower measures of fatty liver. These mechanisms may protect persons with type 1 diabetes from microvascular injury and hyperglycemia-related organ damage. The increased expression of *TDG* and *PARP4* in non-progressors highlights the contributing role of both epigenetic and post-translational modification mechanisms in preventing development of diabetic complications. *OXPHOS* oxidative phosphorylation, *ROS* reactive oxygen species, *UPC2* uncoupling protein 2, *FIS1* mitochondrial fission 1 protein, *NF-kB* nuclear factor kappa B, *TNFR4* tumor necrosis factor-alpha receptor 4, *IL-1* interleukin 1, *TDG* thymine DNA glycosylase, *PARP4* poly(ADP-ribose) polymerase family member 4, *PDR* proliferative diabetic retinopathy, *DKD* diabetic kidney disease, *RP* rapid progressors to PDR and/or DKD, *NP* non-progressors (created with BioRender.com).

Diabetes associated changes in energy metabolism represent systemic processes, which affect most cells in the body including blood cells. Therefore, it is possible to use blood cells to test the hypothesis of genetic regulation or more directly observe the changes in gene expression, which are linked to diabetes-related phenotypes. In the present study, RNA sequencing of blood cells was used to shed light on the mechanisms linked to protection from vascular damage in persons with long-term type 1 diabetes (Fig. 1).

## Results

### Characteristics of patients: non-progressors vs rapid progressors

RNA extraction from the whole blood was used to investigate the transcriptomic differences between persons with long-term type 1 diabetes classified as non-progressors (NP, n = 106) and rapid progressors (RP, n = 26) to microvascular complications (DR and DKD). The clinical characteristics of the groups are shown in Table 1. As previously reported, NPs were characterized by an earlier onset of type 1 diabetes, better glycemic control and insulin sensitivity, more favorable lipid profile and lower measures of fatty liver indices as compared to RPs ^10^.

**Table 1:** Clinical characteristics of the PROLONG study participants.

	NP	RP	p_mw_	p_a_	p_b_
N	106	26	–	–	–
Sex (men %)	43 (41%)	12 (46%)	0.77	–	–
Age at visit (years)	56.64 (10.49)	42.19 (14.33)	5.00e−06	–	–
Age at diagnosis (years)	14.22 (8.76)	19.69 (13.53)	0.11	1.32e−11	2.45e−09
Duration of type 1 diabetes (years)	42.42 (8.84)	22.5 (8.85)	3.42e−12	1.32e−11	2.45e−09
HbA_1c_ (mmol/mol)	61.6 (10.75)	83.62 (18.42)	4.09e−08	9.24e−09	–
HbA_1c_ (%)	7.79 (0.98)	9.8 (1.69)	4.09e−08	9.27e−09	–
BMI (kg/m^2^)	24.89 (4.19)	25.87 (5.62)	0.42	0.66	0.92
HDL (mmol/L)	1.85 (0.58)	1.42 (0.43)	1.02e−04	0.03	0.46
LDL (mmol/L)	2.57 (0.68)	2.78 (0.97)	0.54	0.76	0.32
Triglycerides (mmol/L)	0.78 (0.37)	1.08 (0.4)	1.93e−04	5.92e−04	0.07
Hypertension (%)	53 (50%)	17 (65%)	0.23	5.10e−04	1.85e−03
Retinopathy (%)	0 (0%)	20 (77%)	–	7.88e−34	–
Nephropathy (%)	0 (0%)	10 (38%)	–	7.07e−11	–
Smoking (current %)	4 (4%)	4 (15%)	0.08	0.02	0.07
Waist circumference (cm)	87.43 (12.72)	89.67 (14.66)	0.54	0.29	0.71
Waist/Hip ratio	0.86 (0.08)	0.86 (0.1)	0.7	0.27	0.8
Systolic BP (mmHg)	129.4 (16.07)	126.96 (24.15)	0.61	0.1	0.27
Diastolic BP (mmHg)	87.88 (9.08)	88.91 (12.14)	0.76	0.38	0.59
eGDR (mg/kg/min)	7.29 (2.34)	5.46 (2.69)	1.93e−03	9.69e−06	0.01
HSI^a^	33.4 (5.46)	36.9 (7.63)	0.03	0.06	0.75
FLI^b^	0.57 (0.72)	0.8 (0.74)	0.12	0.03	0.28
eGFR (mL/min/1.73 m^2^)	90.6 (16.0)	99.8 (37.1)	0.08	0.61	0.87
Lipid-lowering treatment	64 (60%)	13 (50%)	0.43	0.13	0.15
Anti-hypertensive treatment	43 (41%)	13 (50%)	0.54	0.003	0.02

*eGDR* estimated glucose disposal rate, *HSI* hepatic steatosis index, *FLI* fatty liver index, *eGFR* estimated glucose filtration rate, *p*_*mw*_ p-value from Mann–Whitney test, *p*_*a*_ p-value from sex- and age-adjusted linear regression, *p*_*b*_ p-value from sex, age and HbA_1c_-adjusted linear regression.

^a^n_NP_ = 105 and n_RP_ = 25.

^b^n_NP_ = 104.

### Differential expression and functional enrichment of blood transcriptome

**Figure 2:**
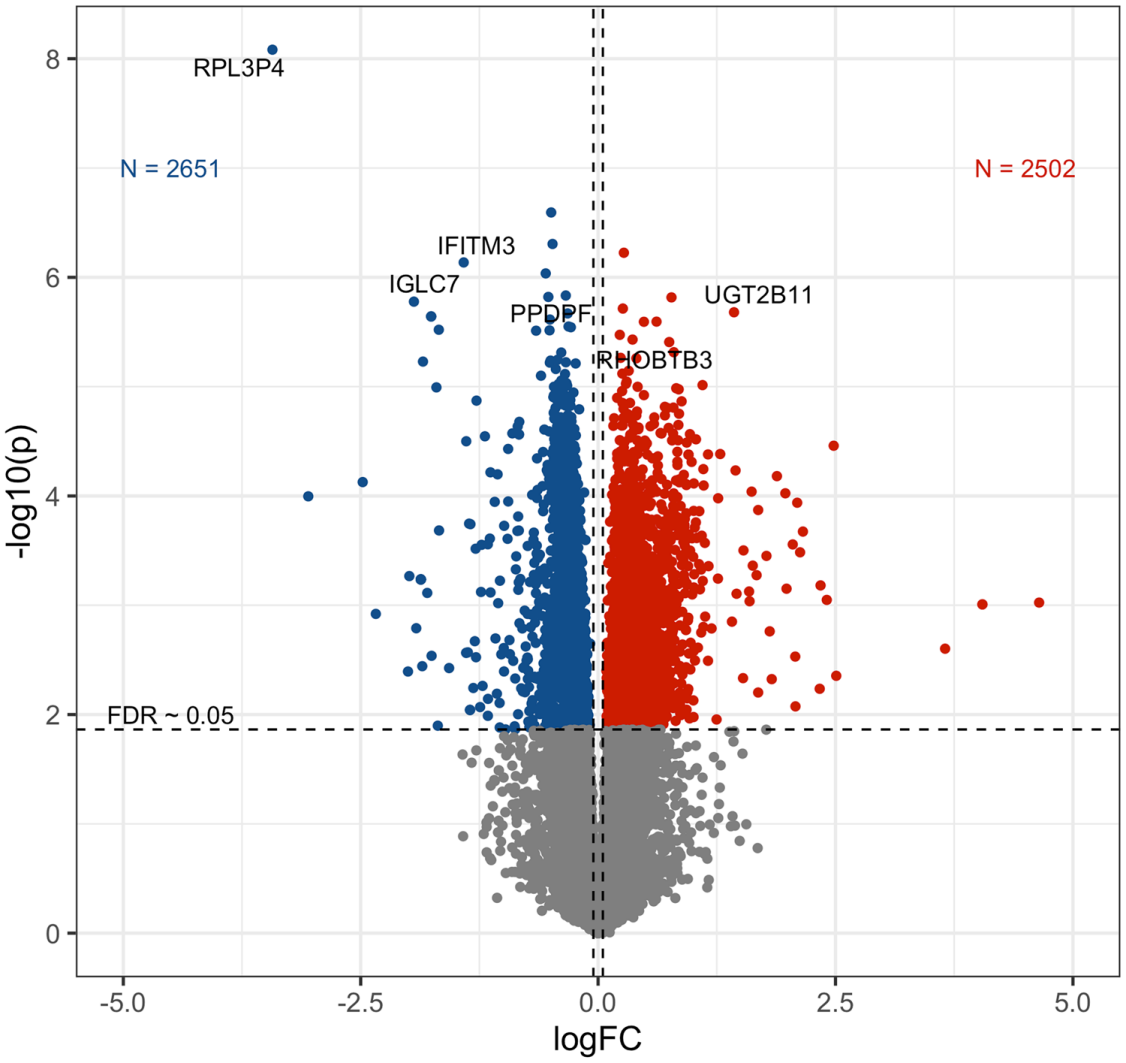
Volcano plot showing variation of significance and log-fold-change for age-adjusted differential gene expression analysis.

A large number of genes was found to be differentially expressed in blood cells between the two groups. A gene was labeled as differentially expressed between the groups after multiple testing adjustment of p-values using false discovery rate (FDR) < 0.05. Overall, there were approximately 5000 differentially expressed genes (DEGs) detected in age-adjusted DE analysis at FDR ≤ 0.05 (Fig. 2). The number of DEGs substantially reduced to 50 genes after including HbA_1c_ as a covariate in the model. The DEGs between NPs and RPs (FDR < 0.05) are shown in Tables S1 and S2.

**Figure 3:**
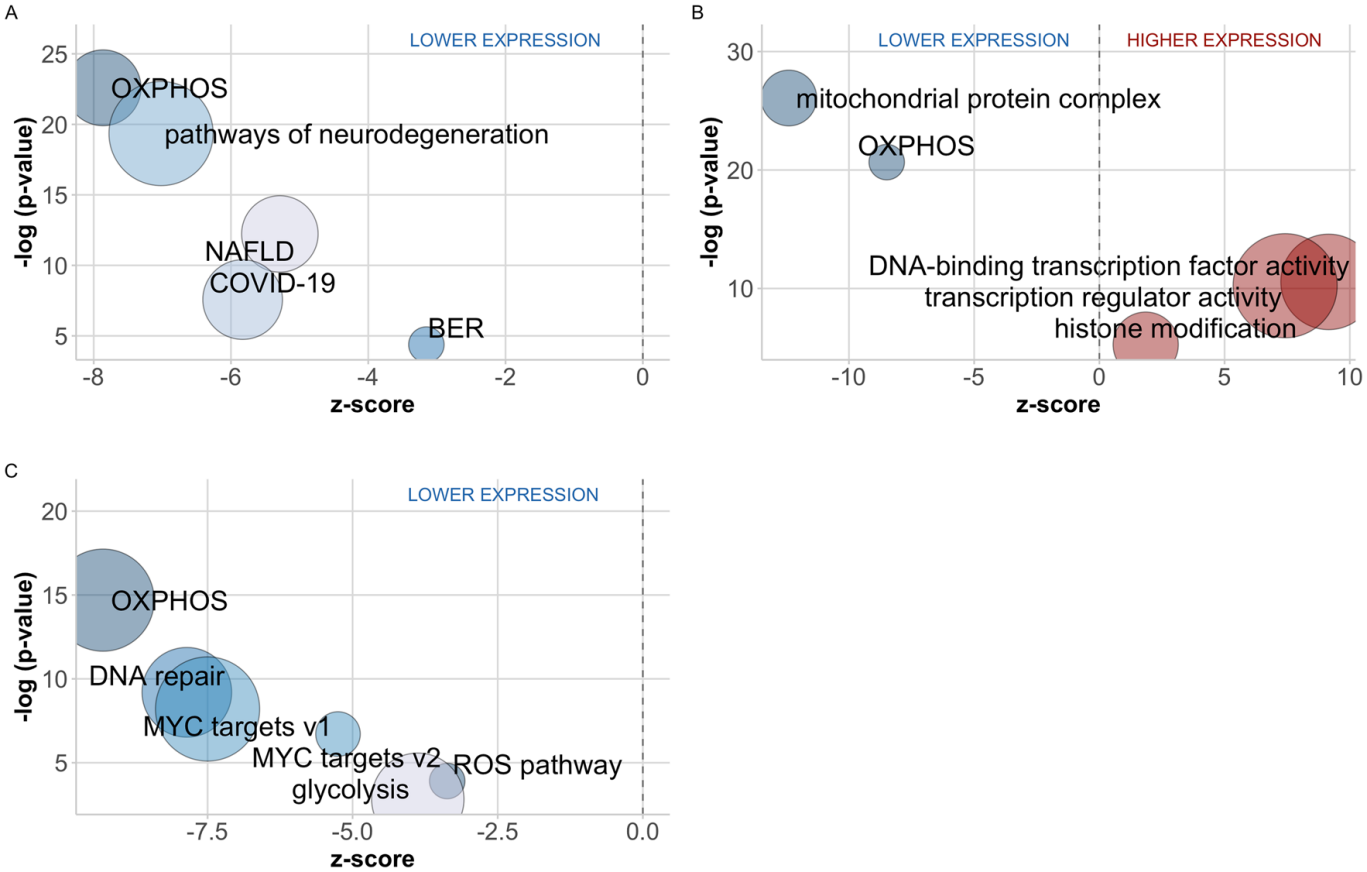
Functional enrichment plots showing variation of statistical significance with z-score based on log-fold change for biological pathway and terms from various databases. **(A)** Over representation analysis results for KEGG pathways, **(B)** over representation analysis results for GO terms, **(C)** gene-set enrichment analysis results (GSEA—HALLMARK genesets). Size of the bubbles are correlated with total number of genes in the pathway. Colors indicate similar pathways. Results are shown for age-adjusted DE analysis.

To provide insights into the underlying biological processes that were associated with the DEGs between NPs and RPs, we performed pathway and ontology term enrichment analyses. The results of over representation analyses for gene ontology (GO) terms in three categories as biological process (BP), molecular function (MF) and cellular component (CC) and KEGG, and gene set enrichment analyses for GSEA gene sets (Hallmark, Reactome and Transcription Factor Targets) are presented in Supplementary Tables S3 to S11 (Fig. 3).

### NPs demonstrated lower expression of OXPHOS and genesets related to mitochondrial function

The results of the over representation analyses using KEGG and GO showed higher number of DEGs in OXPHOS pathway than expected by chance. Additionally, gene set enrichment analysis for GSEA consistently showed that OXPHOS gene expression was overall downregulated in NPs regardless of differential expression. Top enrichment results also consisted of and dominated by mitochondria related terms such as GO oxidative phosphorylation (GO:0006119, p = 1.5 × 10^–22^), GO mitochondrial inner membrane (GO:0,005,743, p = 2.3 × 10^–35^), mitochondrial protein complex (GO:0098798, p = 4.4 × 10^–37^), and GSEA Hallmark geneset for oxidative phosphorylation (p = 2.0 × 10^–15^), which were all enriched for the genes downregulated in NPs (Tables S3–S7). All oxidative phosphorylation sets contained different number of genes with substantial overlap for the canonical part of the pathway, but also had different genes arising from different curation processes in different databases.

Specifically, in the DE analysis (age-adjusted), NPs demonstrated lower expression of the genes encoding the outer and inner mitochondrial membrane translocases (*TOMM6*, *TOMM7, TOMM22, TOMM40, TOMM40L, TIMM13, TIMM17B, TIMM8B, TIMM9, TIMM23*), TCA cycle enzymes (*IDH3B, IDH3G*, *SDHB*, *SUCLG1*, *MDH2, ACO2, PDHA1*), and mitochondrial ribosomal proteins (*MRPL*s) compared to RPs (FDR < 0.05). Enriched KEGG pathways for the downregulated genes in NPs also mostly included mitochondrial function as a part of the main age-related disorders pathway such as Parkinson disease (p = 6.7 × 10^–27^), Huntington disease (p = 2.9 × 10^–24^), thermogenesis (p = 1.7 × 10^–17^), Alzheimer disease (p = 5.0 × 10^–17^), amyotrophic lateral sclerosis (ALS) (p = 7.0 × 10^–21^), pathways of neurodegeneration—multiple diseases (p = 4.5 × 10^–20^) and non-alcoholic fatty liver disease (NAFLD) (p = 5.9 × 10^–13^).

Respiratory electron transport, ATP synthesis by chemiosmotic coupling, and heat production by uncoupling proteins was statistically significant for the direction of downregulation in NPs (Reactome, p = 1.7 × 10^–15^). NPs demonstrated lower level of mitochondrial biogenesis (*FIS1*) and mitophagy (*LAMP1*) regulator expressions as compared to RPs (age-adjusted DE analysis—Table S1). Furthermore, target genes for MYC oncoprotein, which directly involves in the regulation of mitochondrial biogenesis ^14^ were downregulated in NPs (GSEA, MYC_TARGETS_V1, p = 6.3 × 10^–9^, MYC_TARGETS_V2, p = 2.0 × 10^–7^).

Notably, antioxidant response components such as *NFE2, GPX1, GPX4* genes and overall Reactome Detoxification of Reactive Oxygen Species Pathway (p = 0.006), which is a pathway of antioxidant response, were downregulated in NPs. The lower OXPHOS expression in NPs was associated with reduced expression of apoptosis regulators (*BAD, BBC3, BCL2L12, BAX*) and uncoupling protein (*UCP2*) dissociating oxidation and phosphorylation processes. No pathways or terms for apoptosis reached statistical significance.

Additionally, the top terms from gene set enrichment analyses remained the same after HbA_1c_ adjustment (Table S6–S11). The associations between individual genes in canonical OXPHOS pathway (KEGG) with age, duration of diabetes, HbA_1c_, complications type as retinopathy and nephropathy, as well as and BMI are shown in Fig. S4–S12. The change of expression was independent of BMI and age for most of the genes (Fig. S3).

### DNA repair genes are differentially expressed between NPs and RPs

**Figure 4:**
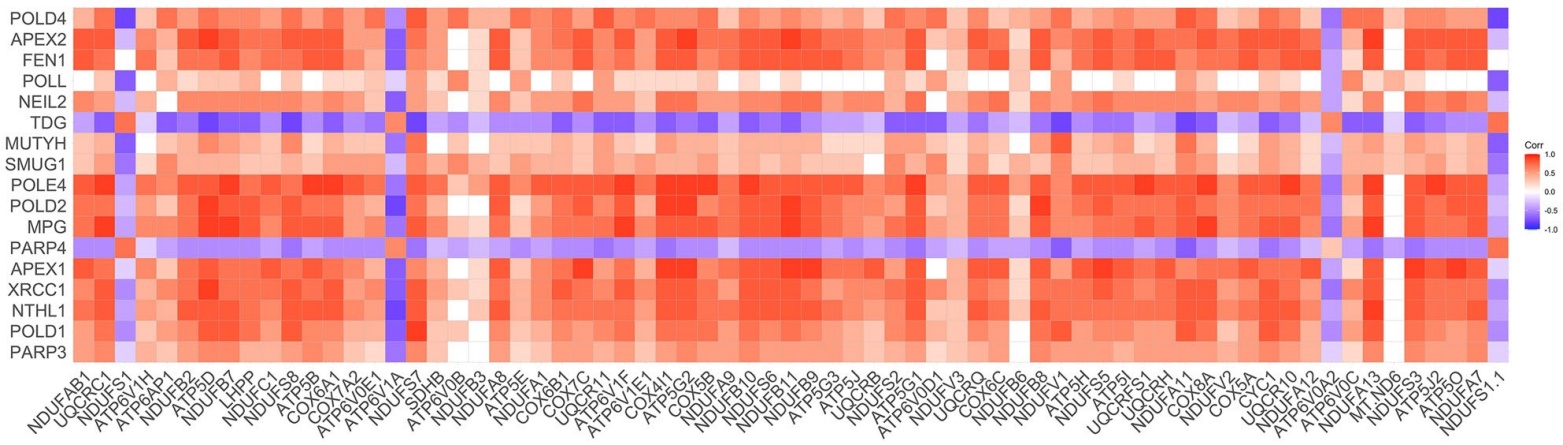
Correlations between differentially expressed OXPHOS (x-axis) and base-excision repair (y-axis) genes.

Another term beyond the dominating mitochondrial genes (domain) was DNA repair (GSEA, p = 6.3 × 10^–10^) (Fig. 3, Table S7) and specifically base-excision repair (BER) (KEGG, p = 4.1 × 10^–5^, Reactome, p = 2.1 × 10^–4^). Expression of genes involved in DNA repair processes was downregulated in NPs. Out of 32 BER genes present in KEGG BER pathway, 50% (15) were downregulated in NPs and positively correlated with OXPHOS genes (Fig. 4). Only 2 genes, poly [ADP-ribose] polymerase 4 (*PARP4*) and thymine DNA glycosylase (*TDG*) were upregulated, and the latter was an exception demonstrating strong negative correlation with OXPHOS and most of the genes encoding mitochondrial enzymes (Fig. 4).

### Moderate reduction of OXPHOS and BER gene expression is associated with better insulin sensitivity and overall metabolic balance

**Figure 5:**
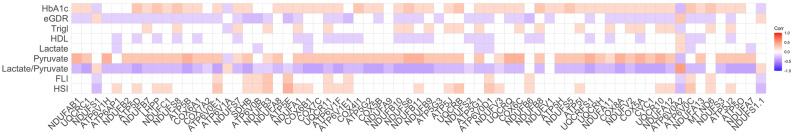
Correlations between differentially expressed OXPHOS genes (x-axis) and metabolic parameters (y-axis).

We have previously demonstrated that NPs were characterized by higher insulin sensitivity as compared to RPs ^10^. In the present study, the top results from the GSEA transcriptional factor targets enrichment analyses identified *HMGA1*—an important regulator of insulin action and function ^15^ being statistically and significantly upregulated in NPs (Table S11). Additionally, differentially expressed canonical OXPHOS genes (KEGG) showed moderate and positive correlation with HbA_1c_, pyruvate, triglycerides, and measures of fatty liver index (FLI) and hepatic steatosis index (HSI) (Fig. 5). The OXPHOS and BER gene expression negatively correlated with estimated glucose disposal ratio (eGDR) and HDL (Fig. 5 and Fig. S2).

### Reduction in the expressions of OXPHOS and DNA repair genes is associated with downregulation of pro-inflammatory activation of blood cells

Since oxidative stress within the cells is associated with DNA damage, it can result in damage-associated molecular pattern (DAMP) release and trigger immune response. Inflammation-related pathway terms from Reactome database, such as Influenza infection (p = 6.2 × 10^–10^), Dectin 1 mediated noncanonical NF-kB signaling (p = 4.9 × 10^–8^), TNFR2 non-canonical NF-kB signaling (p = 3.7 × 10^–7^), Exogenous soluble antigens cross-presentation typical for dendritic cells (p = 1.2 × 10^–7^), Downstream signaling events of B cell receptor (p = 3.4 × 10^–5^) and Interleukin 1 signaling (p = 8.7 × 10^–5^) were all enriched and downregulated in NPs suggesting reduced activation of immune response in NPs (Table S9). Moreover, expression of inflammatory pathway genes that have a role in COVID-19 infection (KEGG, Coronavirus disease—COVID-19, p = 2.6 × 10^–8^) was also decreased (Table S5). This pathway became available after extensive research on COVID-19 during last year and includes many components of immune response, which shows downregulation of general inflammation in NPs. Importantly and consistently, inflammation-related pathways remained significantly overrepresented after HbA_1c_ adjustment also in the GO terms (Table S4).

## Discussion

The pathophysiology of microvascular damage in patients with type 1 diabetes is complex and multifactorial. Among numerous pathogenic factors, chronic hyperglycemia, endothelial dysfunction, and low-grade inflammation are considered to play a critical role in microcirculatory disturbances and development of vascular complications ^16,17^. In physiological situations, there is an equilibrium between the microcirculation and a rate of metabolism in tissues, while this relationship is impaired in the organs affected by diabetes environment exhibiting reduced blood supply (hypoxia) and as a consequence a high metabolic rate and energy consumption. The key findings in the present study revealed synergistic reduction in expression of OXPHOS, DNA repair and genes regulating immune response pathways in persons with long-term type 1 diabetes who remain largely free from vascular complications. Clinically, this was linked to higher insulin sensitivity and overall better metabolic balance in persons who remain free from complications.

OXPHOS is one of the core systems of the metabolic control of cells, which impacts cell survival, growth, proliferation, functioning and death. OXPHOS generates a substantial amount of superoxide derived from electron leaks while substrates are metabolized. Complexes I and III of mitochondrial respiratory chain generate O^2−^ that is quickly transformed by superoxide dismutase (SOD) into H_2_O_2_ with its further neutralized by catalase and glutathione peroxidase ^18^. Therefore, mitochondrial energy release comes with the cost of generation of ROS, and consequently oxidative damage. There is a direct link between the intensity of OXPHOS and ROS production ^19^ and subsequent damaging effects on cells ^20^. ROS produced through OXPHOS have also been implicated in neurodegenerative diseases ^21^. The retinal pigment epithelium was found to be prone to DNA damage due to oxidative stress ^22^. Increase of ROS via mitochondria was found to be an important contributor to the age-related macular degeneration ^23^ and kidney damage and inflammation in persons with diabetes ^24^. However, the evidence of antioxidants’ benefits on DKD progression to end-stage renal failure was inconclusive ^25^. This could partially be attributed to the protective properties of ROS as signalling molecules under normal physiological conditions ^26^. Our present findings are in line with these notions indicating that modest downregulation of OXPHOS may be beneficial to preserve physiological functions of ROS in non-progressors and thereby contributing to organ protection from oxidative stress.

Another role of ROS beyond oxidative cell damage is its genotoxic effect causing DNA damage and altering gene expression ^12^. Repair of damaged DNA involves various mechanisms, including base-excision repair (BER), homologous recombination (HR), nucleotide excision repair (NER), mismatch repair (MMR) and non-homologous end joining (NHEJ) ^27^. BER is considered to be a key pathway for recognition and repair of diabetes-induced persistent ROS–induced DNA damage ^28^. Although the role of OXPHOS-born ROS and DNA damage in the risk of development of diabetic vascular complications is widely recognized, results from the present study illuminate the important crosstalk between mitochondrial transcriptome and the DNA repair machinery in persons with type 1 diabetes resistant to vascular complications. About 40% of the genes encoding BER pathway enzymes were found to be upregulated in RPs and highly correlated with OXPHOS genes. The simultaneous increase in both may cause more DNA damage than dysfunctional OXPHOS alone since it was reported that single-strand breaks can be generated by ineffective BER or oxidative stress ^29^. However, we cannot rule out that increased expression of OXPHOS genes could be a result of DNA damage as a consequence of defective functioning of DNA N-glycosylase proteins ^30^. Besides, a BER-associated DNA-glycosylase, *TDG*, which plays a key role in active DNA demethylation by recognizing and excising 5-formylcytosine (5fC) and 5-carboxylcytosine (5caC) in the CpG sites, and installing an unmethylated cytosine ^31^, was upregulated in NPs and negatively correlated with most of the mitochondrial enzymes. On the contrary, expression of another DNA glycosylase gene, *NTHL1*, which is essential for BER signatures and oxidative damage present in pancreatic cancinomas ^32^ showed reduced expression in NPs. Hence, the OXPHOS and DNA repair enzymes are highly interconnected. Their lower expression in NPs contributing to cellular defense against genetic alterations could protect cells against violation of various biological processes such as cell proliferation, survival and apoptosis, cell signaling and transcription affecting cellular functions.

An additional explanation can reside in the common regulation of both OXPHOS ^33^ and BER genes ^34^ by hypoxia, which is commonly present in diabetes. Hypoxia inducible factor (HIF), which is the main cellular adaptor to hypoxia, is supressed in diabetes ^35^. An impaired response of HIF to hypoxia has been described in diabetes with direct pathogenic role on different complications of diabetes^35^. In perfect agreement, gain-of-function genetic variants in the HIF gene that are more active in the diabetic environment are protective for nephropathy and retinopathy in patients with type 1 diabetes ^36,37^. Notably, HIF is one of the strongest metabolic signals regulating activity of key glycolytic enzyme pyruvate kinase 2 (PKM2). PKM2 is the key rate-limiting enzyme in the last step of glycolysis and thereby determines glycolytic flux. Recent observations from the Joslin Diabetes Center suggested that enhanced metabolism of intracellular glucose via activation of PKM2 can protect from diabetic nephropathy along with preventing or reversing mitochondrial dysfunction dysfunction^38,39^. By interacting with HIF1a—a master regulator of the reaction to hypoxia, PKM2 plays an important role in metabolic shift of glucose metabolism away from oxidative phosphorylation towards a glycolytic program. Importantly, increased glycolysis could be at play serving both bioenergetics and metabolic signaling functions^40^. Thereby, whether NPs have less suppression of HIF activity (functionally through a better glucose control or genetically through a more active HIF ^36,37^) followed by reduction of OXPHOS and BER pathways warrants investigation.

Another important finding was downregulation of KEGG thermogenesis in NPs, which in addition to downregulated OXPHOS, may cause decreased ROS generation due to oxidation and phosphorylation coupling ^41^. Uncoupling proteins are responsible for converting mitochondrial membrane potential to heat production ^42^. In our study *UCP2* expression was significantly lower in NPs comparing with RPs, reflecting the role of uncoupling in the development of diabetic complications. *UCP2*, an ubiquitous form of uncoupling proteins, plays an important role in diabetes as contributes to the mitigation of ROS, regulate glucose sensing and insulin release ^43^. Due to uncoupling, upregulated expression of OXPHOS genes in RPs can be associated with a decrease of mitochondrial ATP generation stimulating alternative sources of energy production. It is well known that diabetes is associated with changes in levels of lactate and pyruvate, and their ratio in the peripheral blood reflects the balance between mitochondrial oxidation and glycolysis ^44^. As previously reported, we observed lower levels of pyruvate in NPs as compared to RPs in this cohort^10^. In the present study, the correlation between differentially expressed OXPHOS genes and pyruvate levels further provide a supportive evidence for an important role of cross-talk between glycolysis and mitochondrial oxidative phosphorylation in the development of vascular complications in persons with type 1 diabetes. Although it is possible that environmental factors might modify this relationship in the present cross-sectional study, another plausible genetic determinant might be genetic intrauterine programming of metabolic responses to hypoxia. Thus, a situation when such a shift of glycolysis and mitochondrial biogenesis has been recognized is during embryonic development whilst trophoblast excluded from the oxygenated maternal blood flow^45^. At this stage, exposure to hypoxia induces stabilization of HIF to support metabolic remodeling towards increased glycolysis acquired during transition of embryonic stem cells^46^. This leads to reduced mitochondrial biogenesis and activation of alternatives to glycolysis, the pentosephosphate- and the sorbitol/polyol auxiliary glucose metabolism pathways—supporting our previous findings in PROLONG^10^ and similar to what has been observed in the T1D Medalists’ kidneys protected from nephropathy^38,39^.

The third affected process seen in the present study was the downregulation of genes in the NAFLD pathway in NPs. These transcriptomics changes are in support of our previous observations of lower measures of fatty liver in NPs compared to RPs ^10^. The key mechanisms of NAFLD are related to insulin resistance and the metabolic syndrome. Our earlier observations of higher insulin sensitivity in NPs ^10^ was further supported by the enrichment of the upregulated *HMGA1*-target gene pathway, which is an important transcription factor in regulation of the insulin receptor. Additionally, as discussed above pyruvate is positively and lactate/pyruvate ratio negatively correlated with OXPHOS and BER genes, further emphasizing the interdependency between metabolism (glycolysis), insulin sensitivity and mitochondrial functioning for protection of DNA damage. It is possible that metabolic shift towards glycolytic program and previously proposed increased nucleotide biosynthesis via pentose-phosphate pathway of glycolysis act as denominator(s) of enhanced glucose metabolism and thereby improved insulin sensitivity and lower accumulation of liver fat in NPs. Furthermore, such DNA damage/repair mechanisms are highly correlated to immune response and inflammation, however, it is important to note that the relationship between DNA repair machinery and immune response is bi-directional ^47^.

Finally, it is important to emphasize a possible contribution of the present findings to the severity and risk of vascular complications in other types of diabetes. Although pathophysiological factors leading to chronic hyperglycemia in different diabetes subgroups (type 1 and type 2 diabetes, MODY) are distinct, metabolic features such as degree of insulin sensitivity/resistance and fatty liver parameters might underpin common mechanisms related to progression towards or free from vascular complications. In support of this, recent observation of novel class of glucose-lowering agents—inhibitors of SGLT2—known to improve insulin sensitivity by inhibiting glucose re-absorption in the kidney, shown to be effective not only in patients with type 2 diabetes but also in type 1 diabetes^48^ and associated with reduced CKD in non-diabetic population^49^.

### Limitations

The analysis was done for cross-sectional transcriptomic measurements. Hence, we could observe association between the forementioned ontology terms/pathways with complication status, but we were not able to study temporal effects neither detect causal relationships for the development of vascular complications. Nevertheless, despite differences in age and HbA_1c_ between non-progressors and rapid progressors, the main associations between OXPHOS, DNA repair and inflammation-related pathways remain consistently and statistically significant after correcting for these factors as covariates in FDR analyses suggesting independent and robust associations. However, cross-sectional study design only depicts one-time measurements of HbA1c, and do not reflect severe impact of chronic hyperglycemia, which is often described as glycemic memory. Legacy of glycemic memory exhibit long-lasting effects and therefore longitudinal analyses of HbA1c effects would be of high value.

## Conclusion

Progression to vascular complications in patients with diabetes is a systemic process, which might be monitored by use of transcriptomic alterations in peripheral blood cells. Synchronous elevation of expression of OXPHOS and DNA repair genes may serve as a hallmark of mitochondrial dysfunction associated with increased ROS production, chronic inflammation and elevated lactate-to-pyruvate ratio levels. In contrast, genetically determined moderate reduction in oxidative metabolism due to possible uncoupling towards enhanced glycolytic program in NPs could be linked to lower DNA damage and lower inflammatory activation of blood cells, and overall better insulin sensitivity. Taken together, present findings suggest that reduction in OXPHOS function might be beneficial by protecting against development of vascular complications in patients with long-term type 1 diabetes.

## Research design and methods

### Study participants

Information on the PROLONG eligibility criteria and complication status was based on initial electronic hospital records ^50^. Recruitment of the participants was initiated at the Scania University Hospital, Malmö, Sweden, in February 2011. The recruitment continued at the Karolinska University Hospital, Danderyd Hospital and South Hospital in Stockholm, Sunderby Hospital in Luleå, University Hospital of Umeå, Sahlgrenska University Hospital in Gothenburg, all in Sweden ^10^. The last PROLONG study visits were conducted at the SDCC in 2015. Persons with T1D were classified based on their diabetes complication status. Non-progressors (NPs) were defined as those with diabetes duration of more than 30 years and who did not develop any major complications (diabetic nephropathy, proliferative retinopathy, myocardial infarction, stroke or chronic foot ulcer), whereas rapid progressors (RPs) were defined as those, who developed any of the above-mentioned complications within 25 years of diabetes duration. NPs who prior to or at the PROLONG study visit were diagnosed with any of the above-mentioned complications were designated “late-progressors” and excluded from the present analyses.

PROLONG study was approved by the local ethics committees (PROLONG-Sweden, Regional Ethics Review Board, Department 1, Lund, Sweden, Dnr 777/2009, PROLONG-Denmark, The Capital Region Ethics Committee, Hillerød, Denmark, Dnr H-2-2013-073, for data analysis of the study in UiB, West regional health authority, Norway, REK-2019/1324) and conducted in accordance with local institutional and national regulations.

### Definition of diabetic complications

Diabetic nephropathy was defined as the presence of (1) macroalbuminuria ≥ 200 µg/min in a timed overnight urine collection, (2) an albumin/creatinine ratio > 300 mg/g as macroalbuminuria, or based on a documented diagnosis of diabetic kidney disease, which is diagnosed by using eGFR < 60 mL/min. Proliferative diabetic retinopathy was assessed with fundus photography and defined as the presence of proliferative retinopathy in at least one eye and/or laser therapy (panretinal photocoagulation), or non-traumatic blindness.

### Procedures and measurements

In the PROLONG study, participants fasted overnight (> 8 h) and were instructed not to take their medication in the morning of the visit. Trained diabetes research nurses and biomedical analysts performed the physical examination following standard operating procedures. On the day of the examination, a signed informed consent was obtained, and blood and urine samples taken. The participants were also asked to fill out a detailed questionnaire. These self-reported records were then validated by the diabetes nurse at the clinical research visit and this information was stored in a database at the Scania University Hospital, Malmö, Sweden.

### RNA sequencing

TruSeq stranded Total RNA kit from Illumina was used for sequencing library generation. Sequencing was done on NextSeq 500 sequencing machine to create paired-end files. The sequencing was done at Lund University, Malmö, Sweden.

### Cell content comparison

The whole blood content of the subjects was compared to in the cell counts from laboratory to check whether the blood cell content differ between two groups. The available cell counts for leukocytes and lymphocytes for the patients (87% of NPs and all RPs) were used to compute difference of cell fractions in the samples. It was found that both lymphocyte and leukocyte percentages in total of the blood cells did not differ significantly between groups (p > 0.5). We used two local tools to compare the cell contents of two groups based on microarray expression signatures for whole blood to confirm this (Fig. S13–S14). DeconRNASeq was used with the signature coming from ref. ^51^ and quanTiseq was used with its internal signature ^52,53^. Both signatures consistently showed no significant differences between cell contents across two groups, except natural killer (NK) cells, which seems to be slightly higher in NPs. Both signatures were created from microarray expression data and conversion from microarray ids to gene ids for the comparison of our own RNA sequencing results was not complete due to duplicative or non-mapping between two id types.

### Processing and analysis of RNAseq data

Total sample size was 154 and the average library size for the included samples were 29.6 ± 15.2 M. The quality of paired-end RNAseq files were checked with MultiQC v1.0 by using fastq files. 22 samples were failed QC and removed from further analysis. Phred score greater than 30 was achieved for each sequencing position for all other samples. There was no adapter contamination shown in QC (adapter contamination < 0.1%), so trimming was not applied in order not to lose information. The alignment of transcripts was done by using Kallisto v0.43.1 ^54^ with GRCh38.p10 reference assembly with Ensembl Homo sapiens v90 annotation as the reference transcriptome ^55^. The downstream analysis from this level was done by using R statistical environment^56^ and R Studio (https://www.rstudio.com). The estimated count values of the transcripts given as Kallisto output were converted to the gene level expression values by using Tximport v1.6.04 ^57^. The genes that had low counts were excluded from the expression matrix with the inclusion criteria of CPM higher than 0.5 for at least the smallest number of the sample subset (n = 34). The differential expression analysis was done with using edgeR v3.20.73 ^58^. The raw count values were provided to edgeR since the tool handles with the normalizations for sequencing depth, gene length and RNA composition of the libraries (TMM normalization). edgeR handles unbalanced sample sizes robustly and power of the analysis does not depend on the ratio of sample sizes. The condition is used as explanatory variable in a quasi-likelihood negative binomial generalized log-linear model (glmQLFit function) with using age and/or HbA_1c_ as covariates. Empirical Bayes quasi-likelihood F-tests were used to assess the differential expression (glmQLFTest function). Sex adjustment was not done via adding sex as an explicit covariate since Robust algorithm was used to down-weight sex-linked genes. False discovery rate (FDR) correction was done by using Benjamini–Hochberg method within the package. The gene was considered differentially expressed after adjustment for multiple testing FDR ≤ 0.05. 5153 genes were found to be differentially expressed for age-adjusted differential expression analysis (610 of them with at least 50%-fold-change, Fig. 2, Fig. S1) while the number is reduced to 49 if HbA_1c_ adjustment is applied alongside age (38 genes with at least 50%-fold-change) with false discovery rate of 0.05. There was no missing data for the variables that are included into this analysis.

Average age of NPs was higher than the RPs by study design. Hence, we checked to see whether there is any difference in the gene expression strongly affected by the age difference between two groups. We used an interaction term between age and complication status (NP and RP) to catch this effect, if there was any, in addition to the other covariates. The result showed a significant change in the expression for only two genes (*RPL3P4* and *CXCL10*) were significant for the interaction between age and complication status, which indicates that the effect of the complication status did not vary as a function of age for the expression of the majority of the genes. For the full design formula of age + condition + age:condition, only *RPL3P4*, *HLA-DRB4*, *MAYDM* and *H19* genes became significant for the age term (FDR < 0.05). The expression of the last three genes increased with age while the first one decreased.

### Functional enrichment analysis

GO and KEGG pathway enrichments were done by using goana and kegga functions ^59^. This was done as an overrepresentation analysis that determined over-represented/enriched biological processes and/or pathways in the list of differentially expressed genes. The genes in the relevant GO terms were extracted from gene ontology (https://www.geneontology.org) and KEGG (genome.jp) databases ^60,61^. GSEA enrichment tests were done by using hallmark, all transcriptional factor targets and Reactome genesets and camera test ^62^ (https://www.gsea-msigdb.org) and employing camera function of edgeR. Gene set enrichment test is the procedure to find any coordinated changes related to a gene set in the current list of genes without taking significance of differential expression into consider. The relevant statistic, which is fold-change in the comparison of gene expression, is aggregated to find the coordinated behavior related with the gene set. Enrichment tests were performed in Jan 2021.

Figures 2, 3, 4 and 5 were generated by using R Studio.

## Supplementary Information

Supplementary Figures.

Supplementary Tables.

## Data Availability

All data is available upon reasonable request to the corresponding author.
