# Supplementary material for: Reduced expression of OXPHOS and DNA damage genes is linked to protection from microvascular complications in long-term type 1 diabetes: the PROLONG study
Source: Sci Rep. 2021 Oct 20;11:20735. doi: 10.1038/s41598-021-00183-z (PMC8528906; doi:10.1038/s41598-021-00183-z)
Supplement: Supplementary file 1 — Supplementary Figures. [file 41598_2021_183_MOESM1_ESM.pdf]

Table S1. List of differentially expressed genes in age-adjusted differential expression analysis (FDR < 0.05)
Table S2. List of differentially expressed genes in age and HbA1c-adjusted differential expression analysis (FDR < 0.05)
Table S3. List of GO terms from over-representation analysis of age-adjusted differential expression results (Multiple testing significance below p = 2.4e-06 for 20978 GO terms)
Table S4. List of GO terms from over-representation analysis of age and HbA1c-adjusted differential expression results (Multiple testing significance below p = 2.4e-06 for 20978 GO terms)
Table S5. List of KEGG pathways from over-representation analysis of age-adjusted differential expression results (Multiple testing significance below p = 0.00015 for 339 KEGG pathways)
Table S6. List of GSEA hallmark gene sets from gene set enrichment analysis of age-adjusted differential expression results (FDR < 0.05)
Table S7. List of GSEA hallmark gene sets from gene set enrichment analysis of age and HbA1c-adjusted differential expression results (FDR < 0.05)
Table S8. List of Reactome pathways from gene set enrichment analysis of age-adjusted differential expression results (FDR < 0.05)
Table S9. List of Reactome pathways from gene set enrichment analysis of age and HbA1c-adjusted differential expression results (FDR < 0.05)
Table S10. List of all transcription factor targets gene sets from gene set enrichment analysis of age-adjusted differential expression results (FDR < 0.05)
Table S11. List of all transcription factor targets gene sets from gene set enrichment analysis of age and HbA1c-adjusted differential expression results (FDR < 0.05)

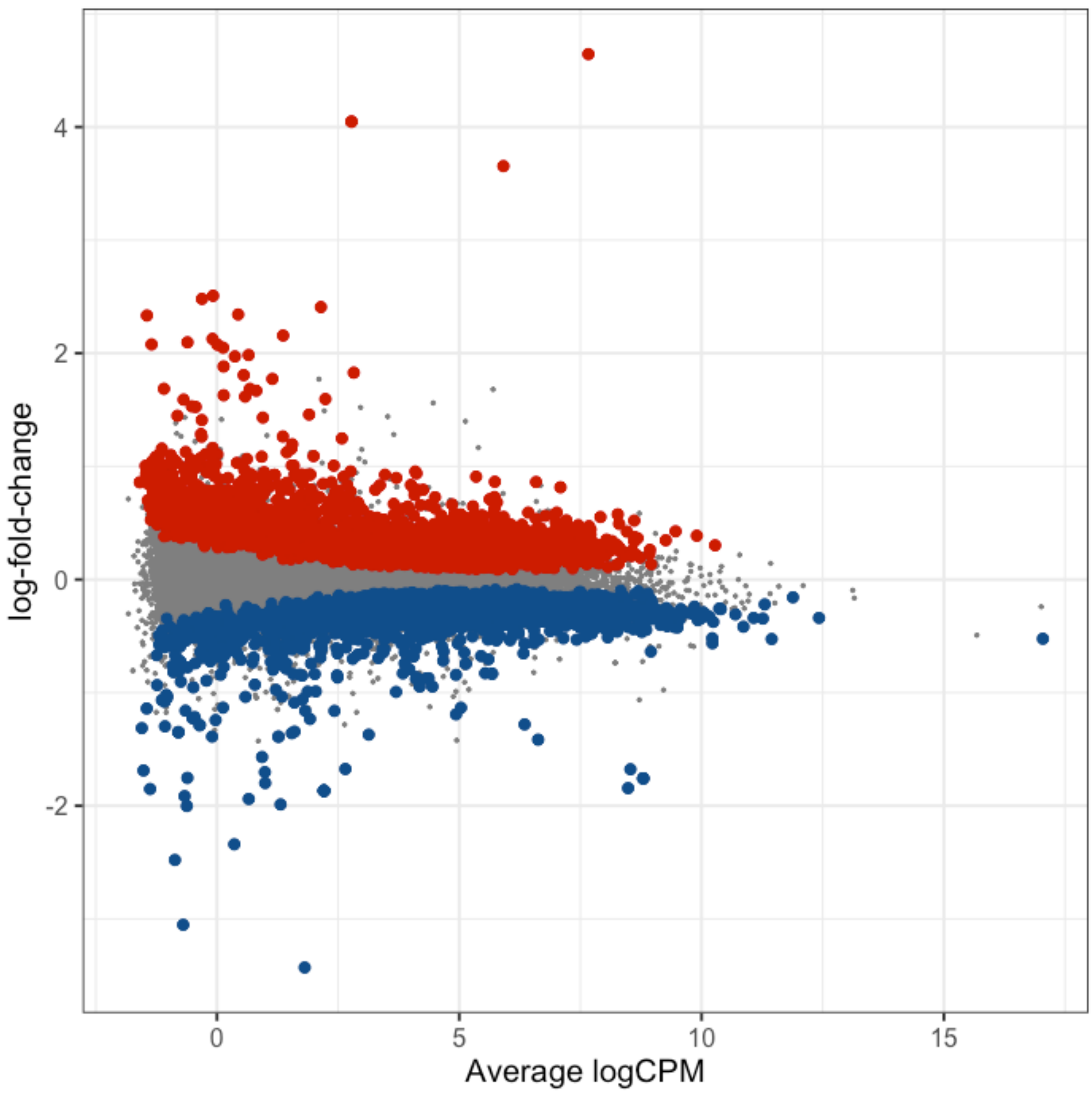

Figure S1. Expression versus fold-change (red points show upregulated and blue points show downregulated genes with FDR ≤ 0.05)

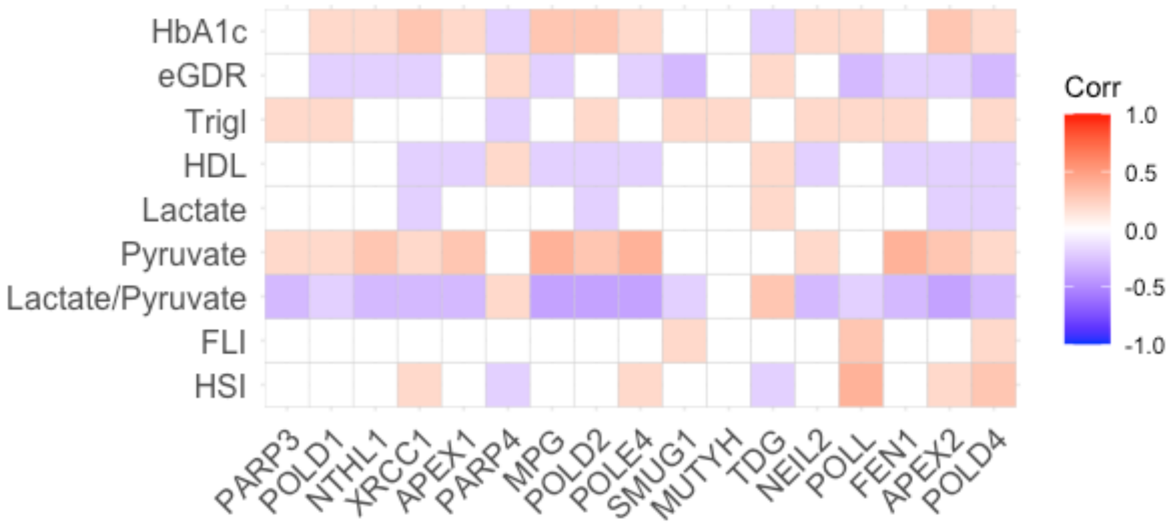

Figure S2. Correlation between BER genes and clinical parameters/measurements

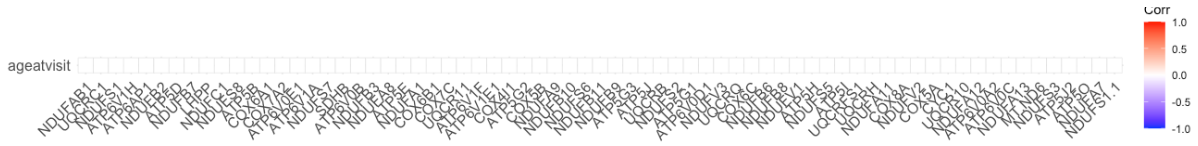

Figure S3. Correlation between OXPHOS genes and age (no gene has significant correlation with age)

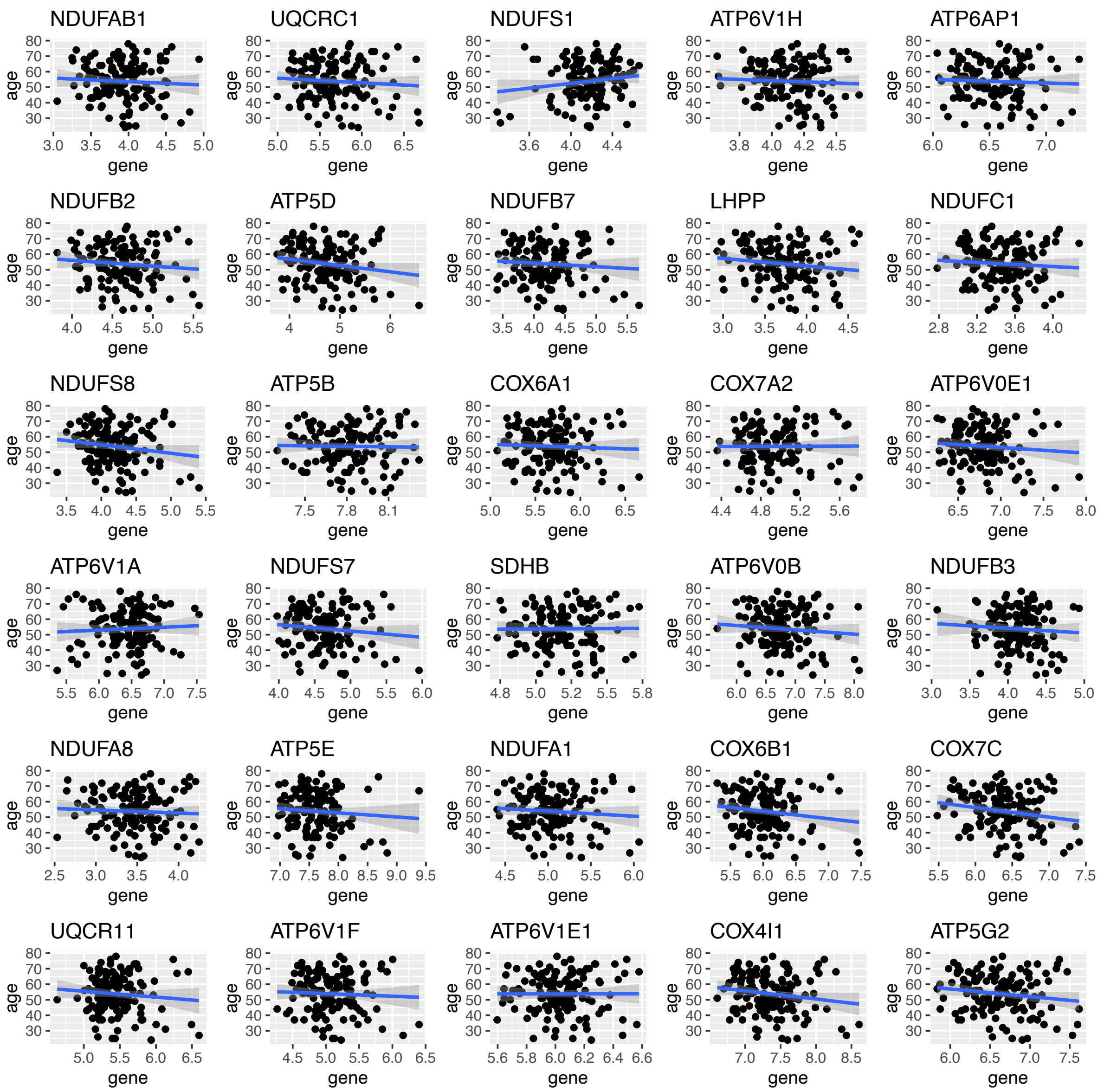

Figure S4a. OXPHOS gene expression versus age for all genes in the pathway

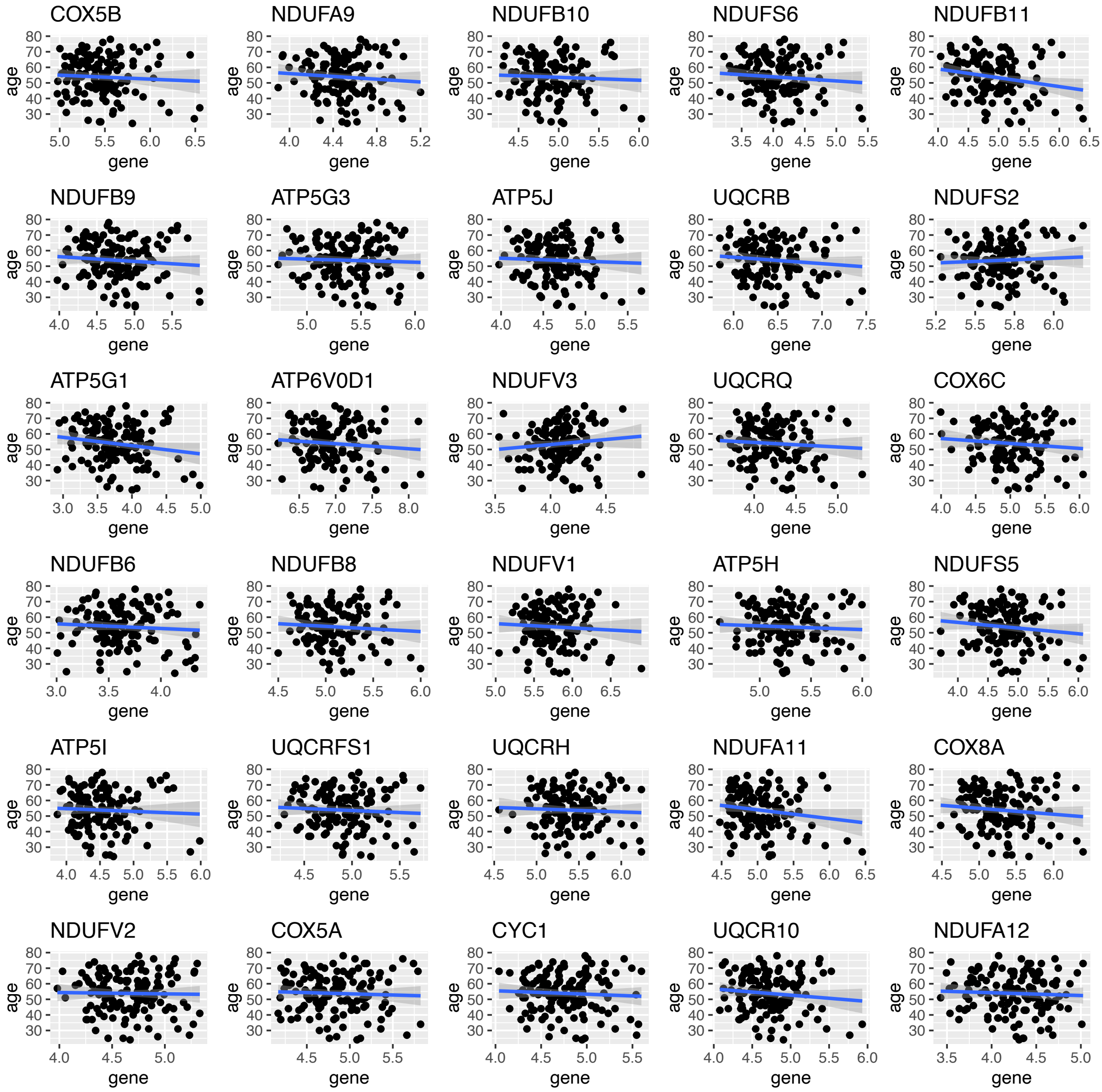

Figure S4b. OXPHOS gene expression versus age for all genes in the pathway

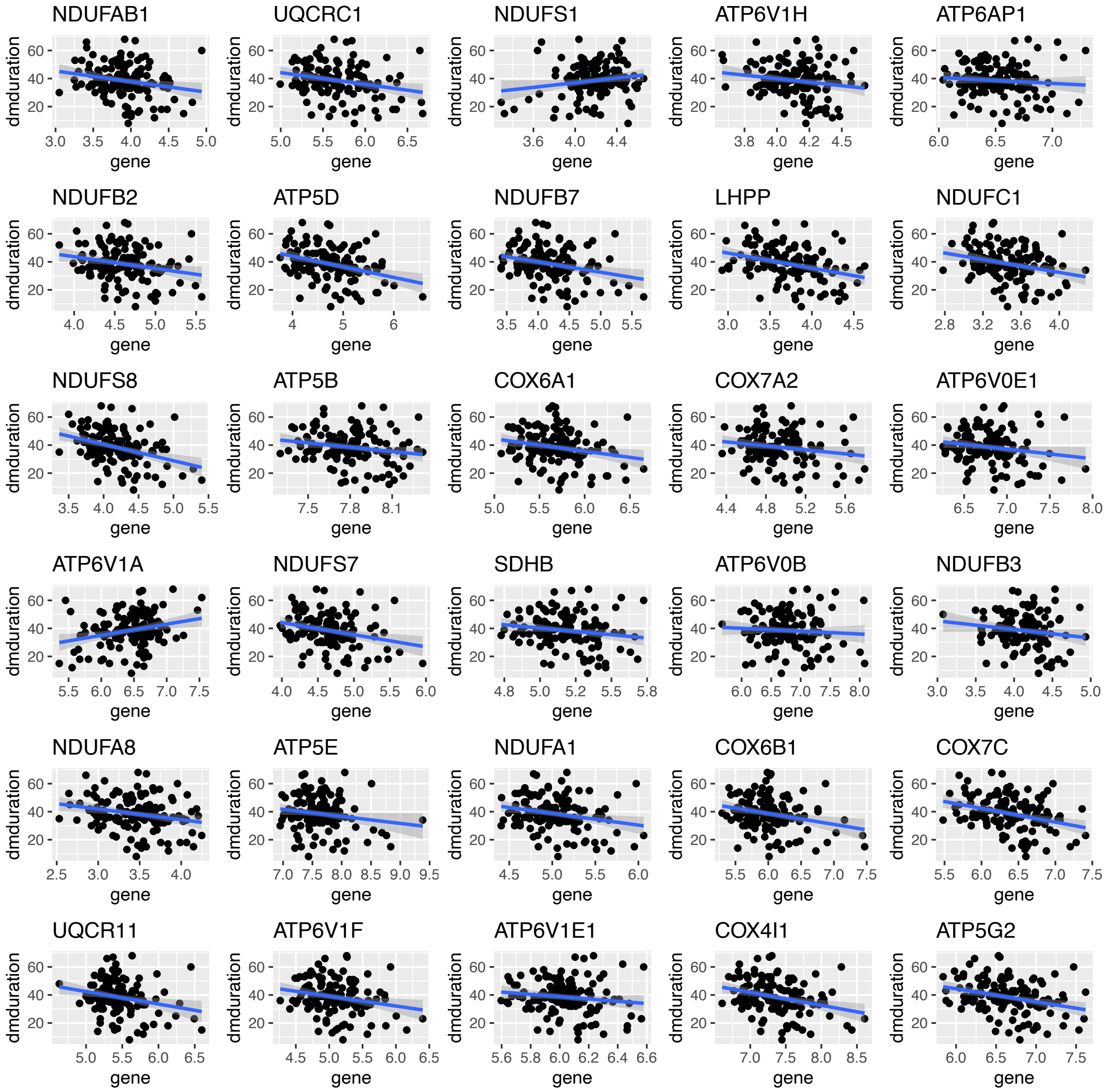

Figure S5a. OXPHOS gene expression versus duration of diabetes for all genes in the pathway

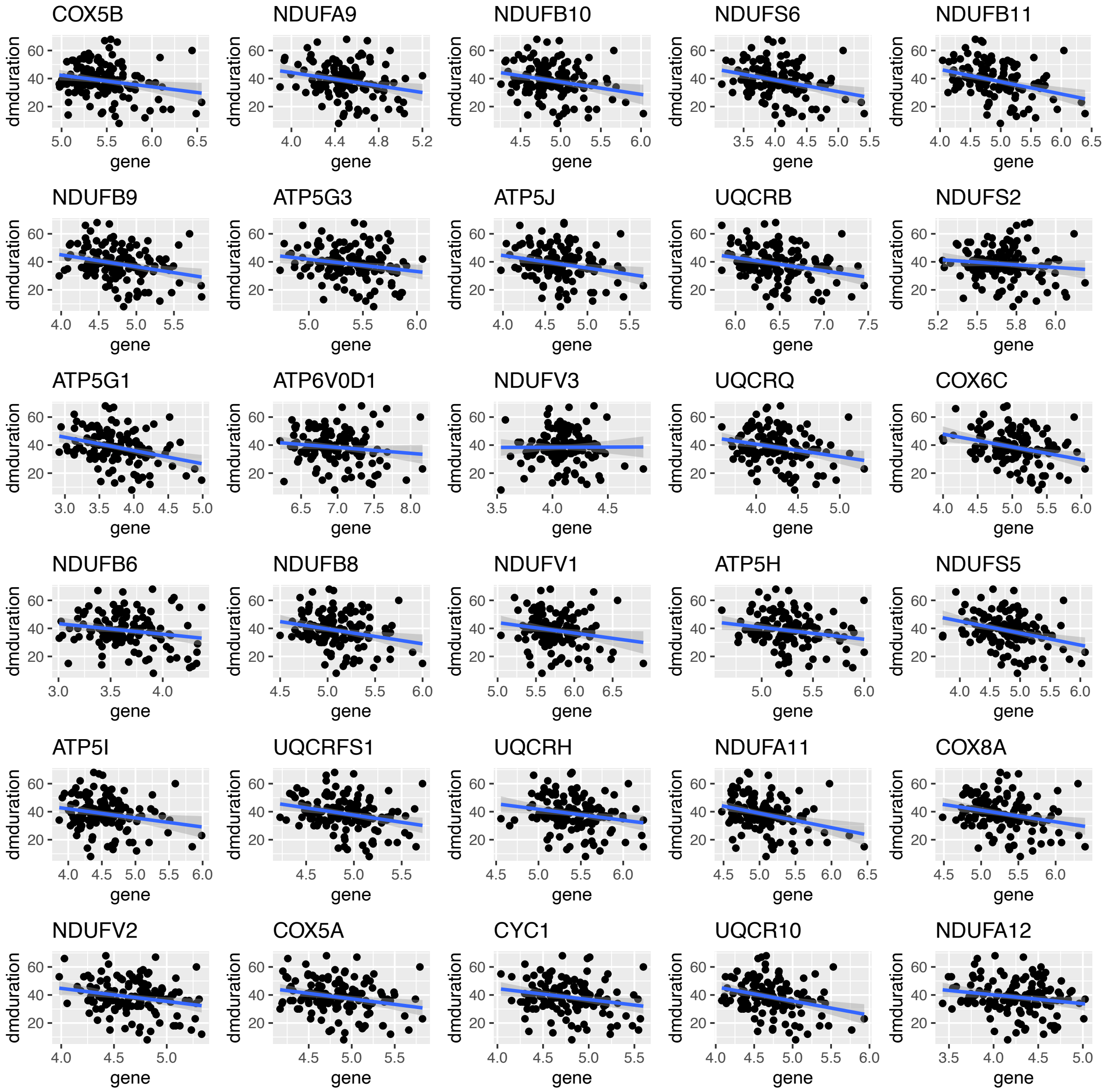

Figure S5b. OXPHOS gene expression versus duration of diabetes for all genes in the pathway

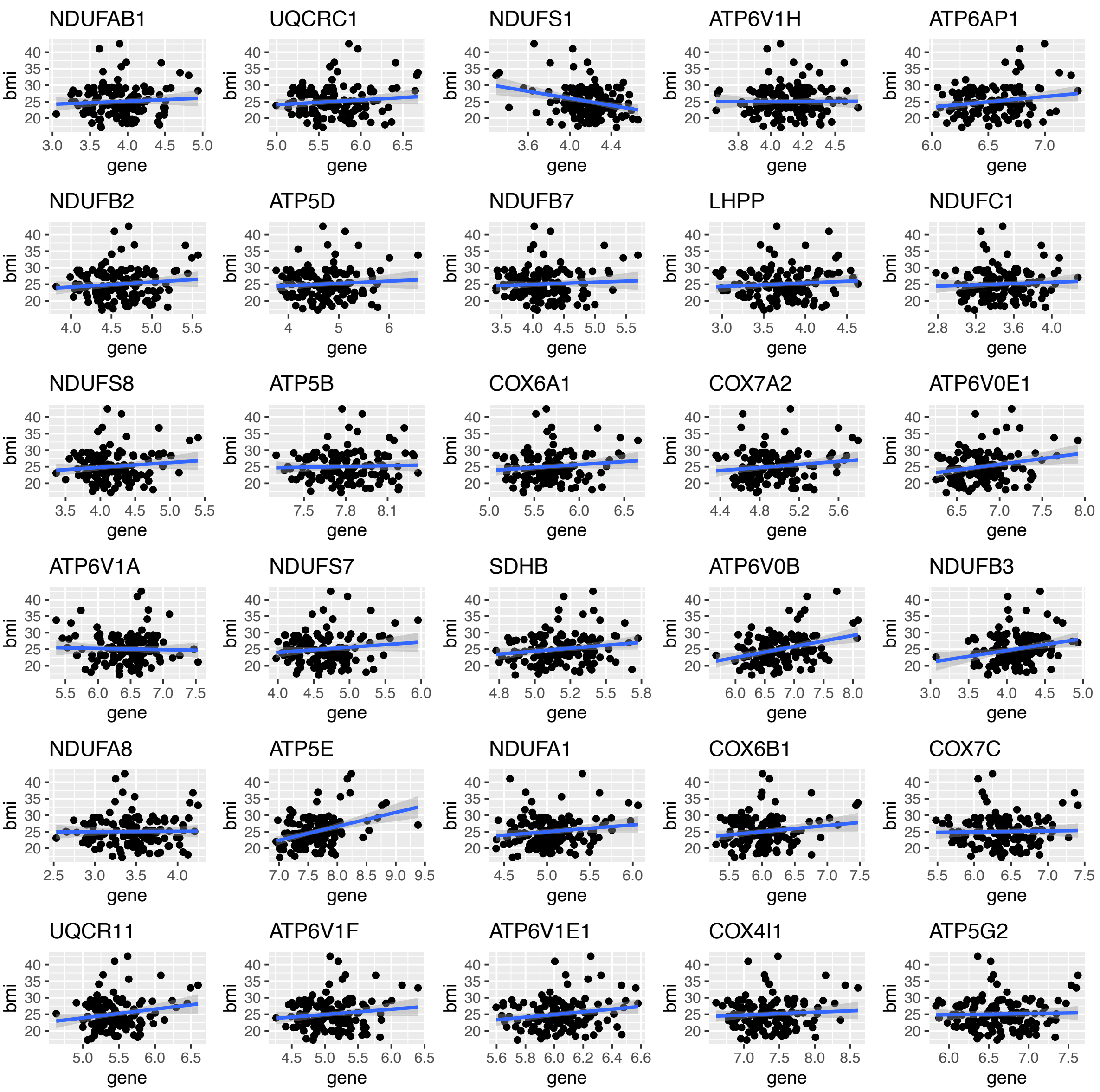

Figure S6a. OXPHOS gene expression versus BMI for all genes in the pathway

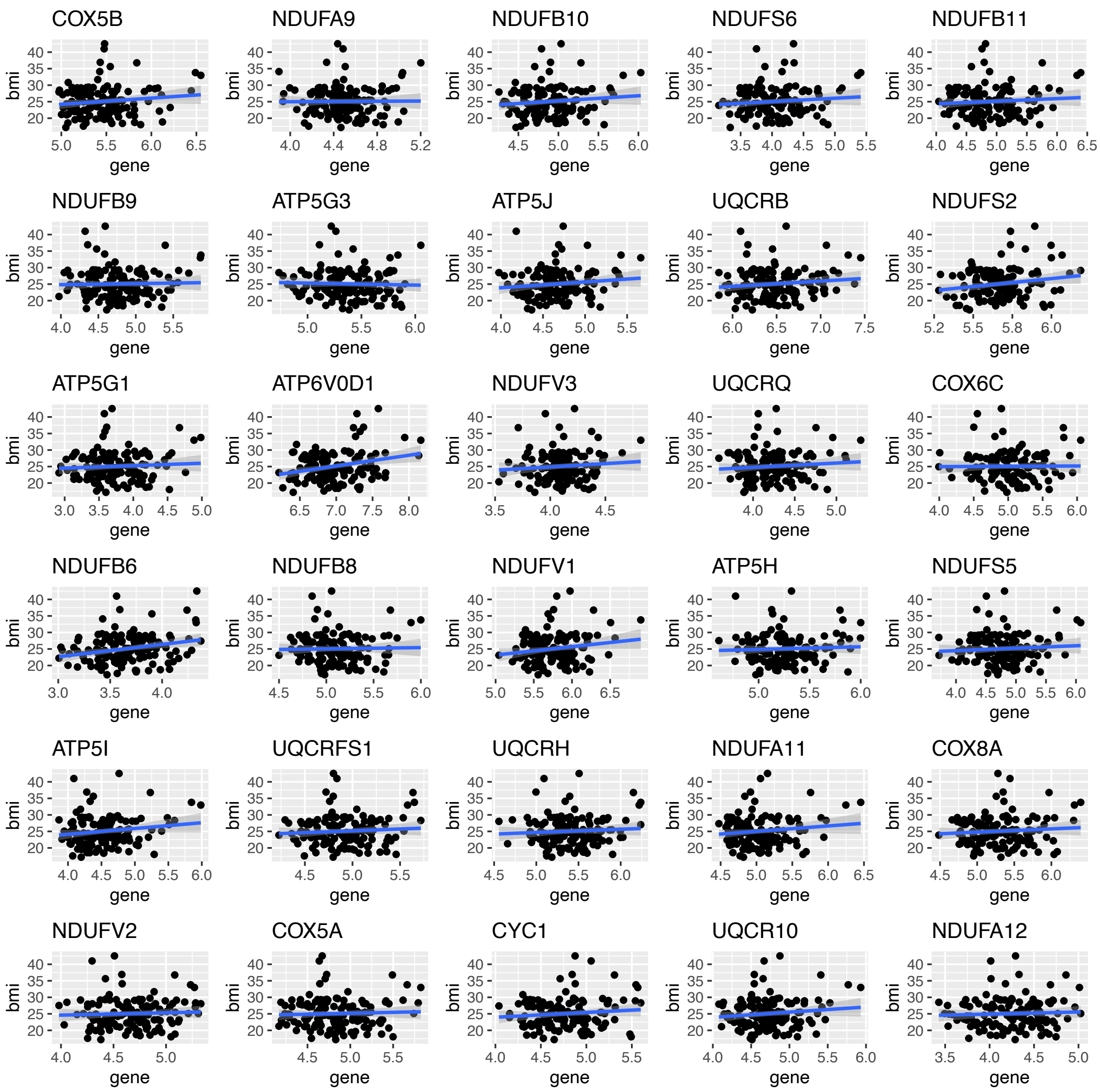

Figure S6b. OXPHOS gene expression versus BMI for all genes in the pathway

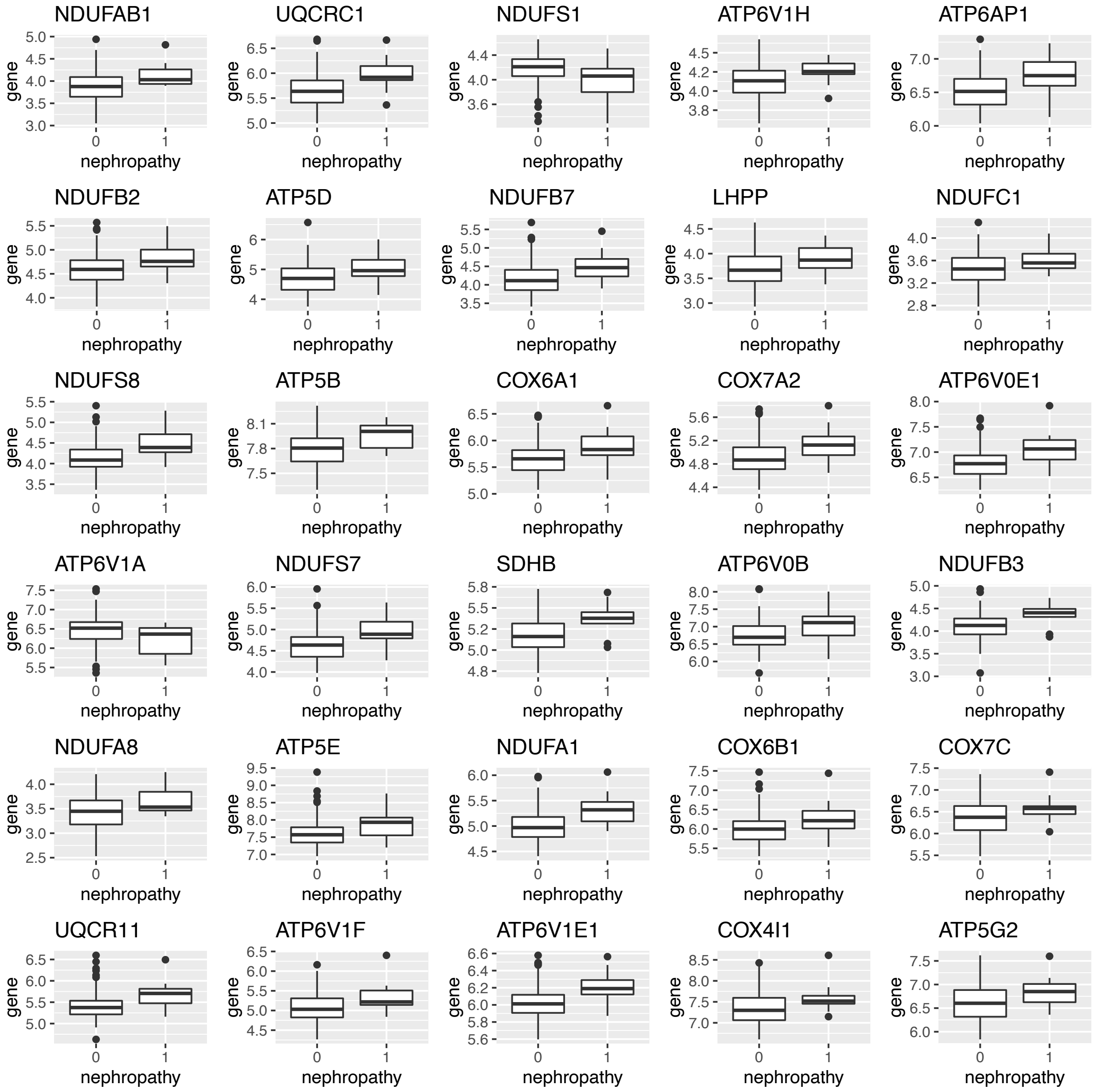

Figure S7a. OXPHOS gene expression changes between nephropathy/non-nephropathy for all genes in the pathway

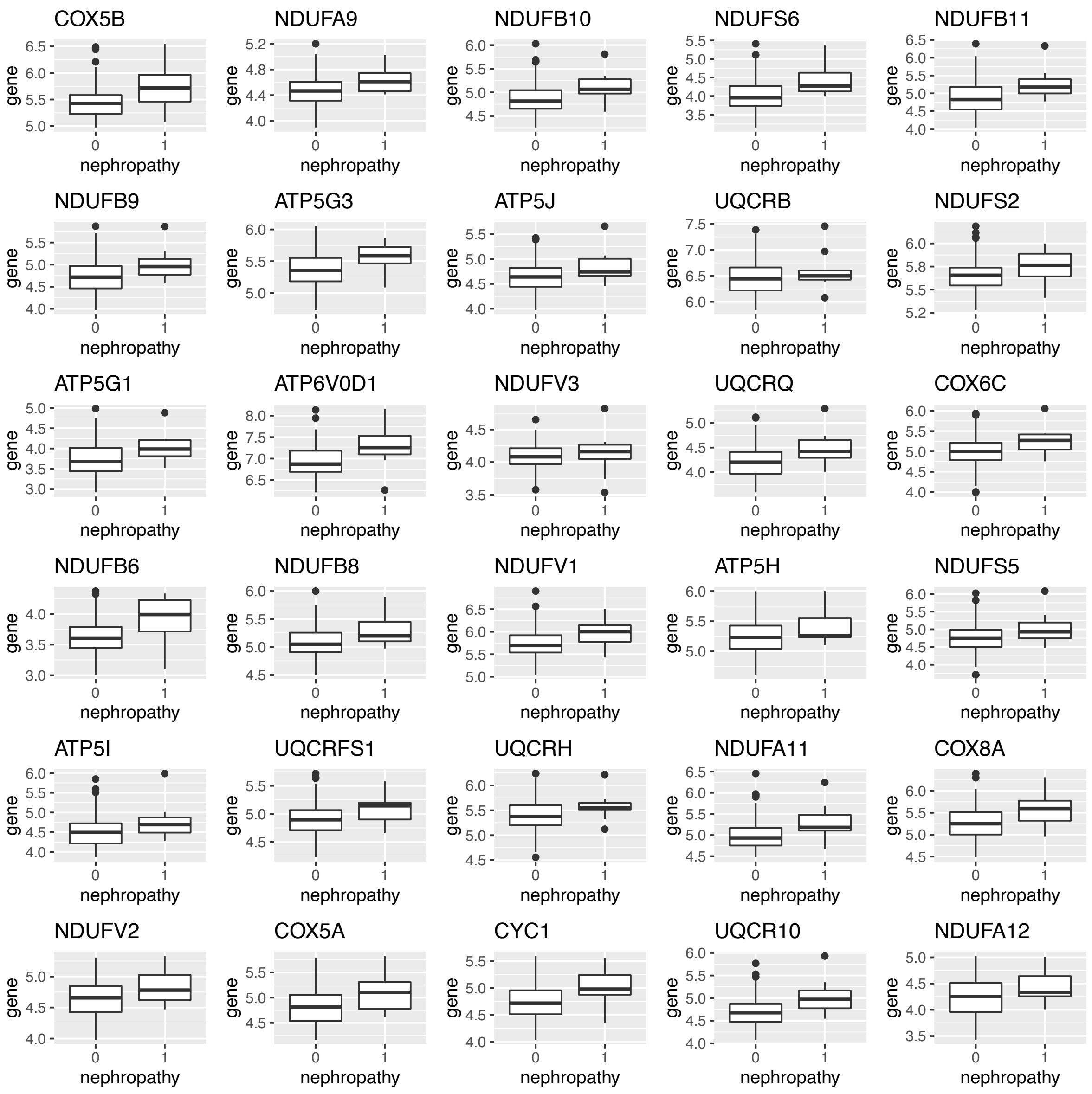

Figure S7b. OXPHOS gene expression changes between nephropathy/non-nephropathy for all genes in the pathway

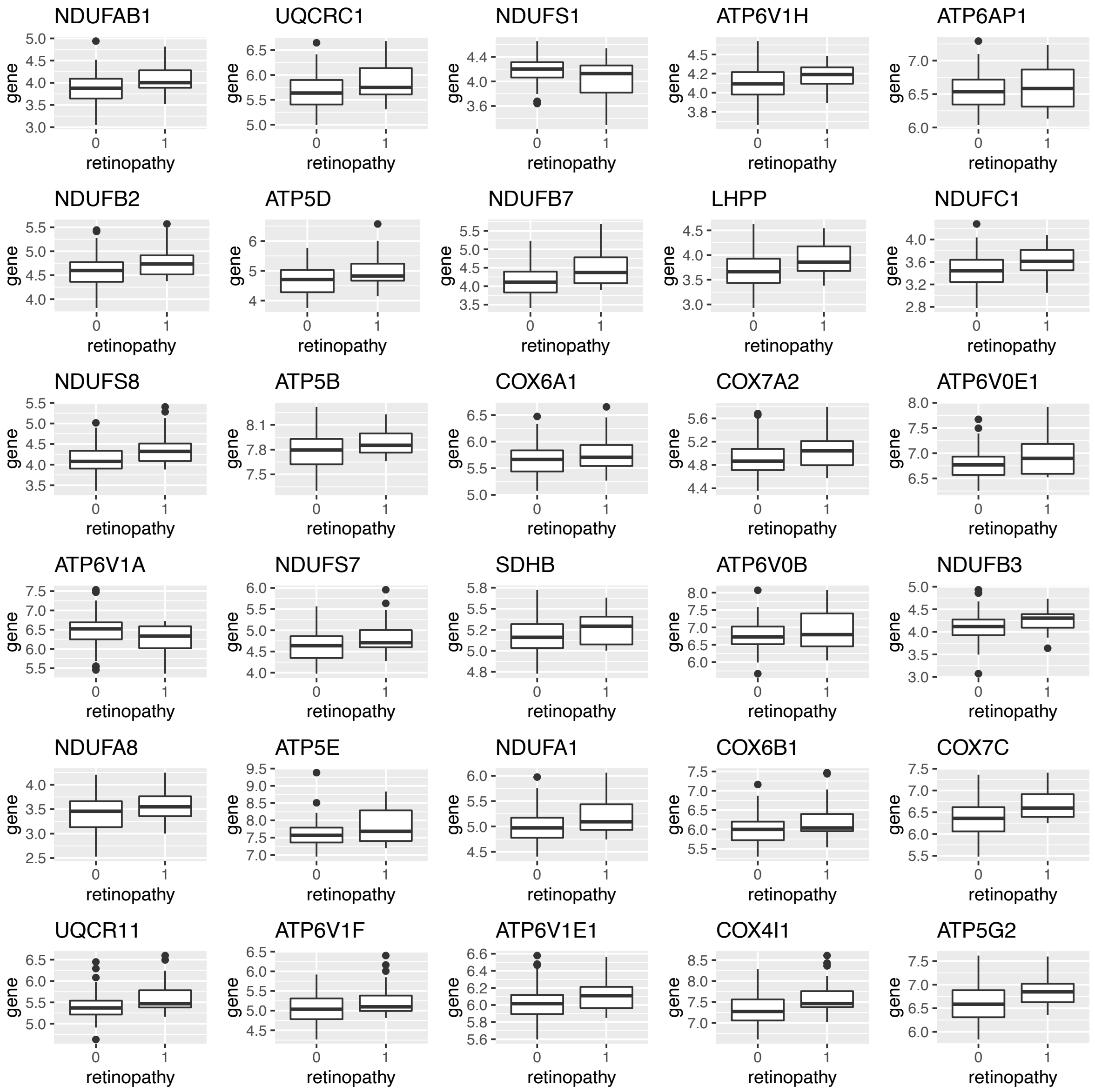

Figure S8a. OXPHOS gene expression changes between retinopathy/non- retinopathy for all genes in the pathway

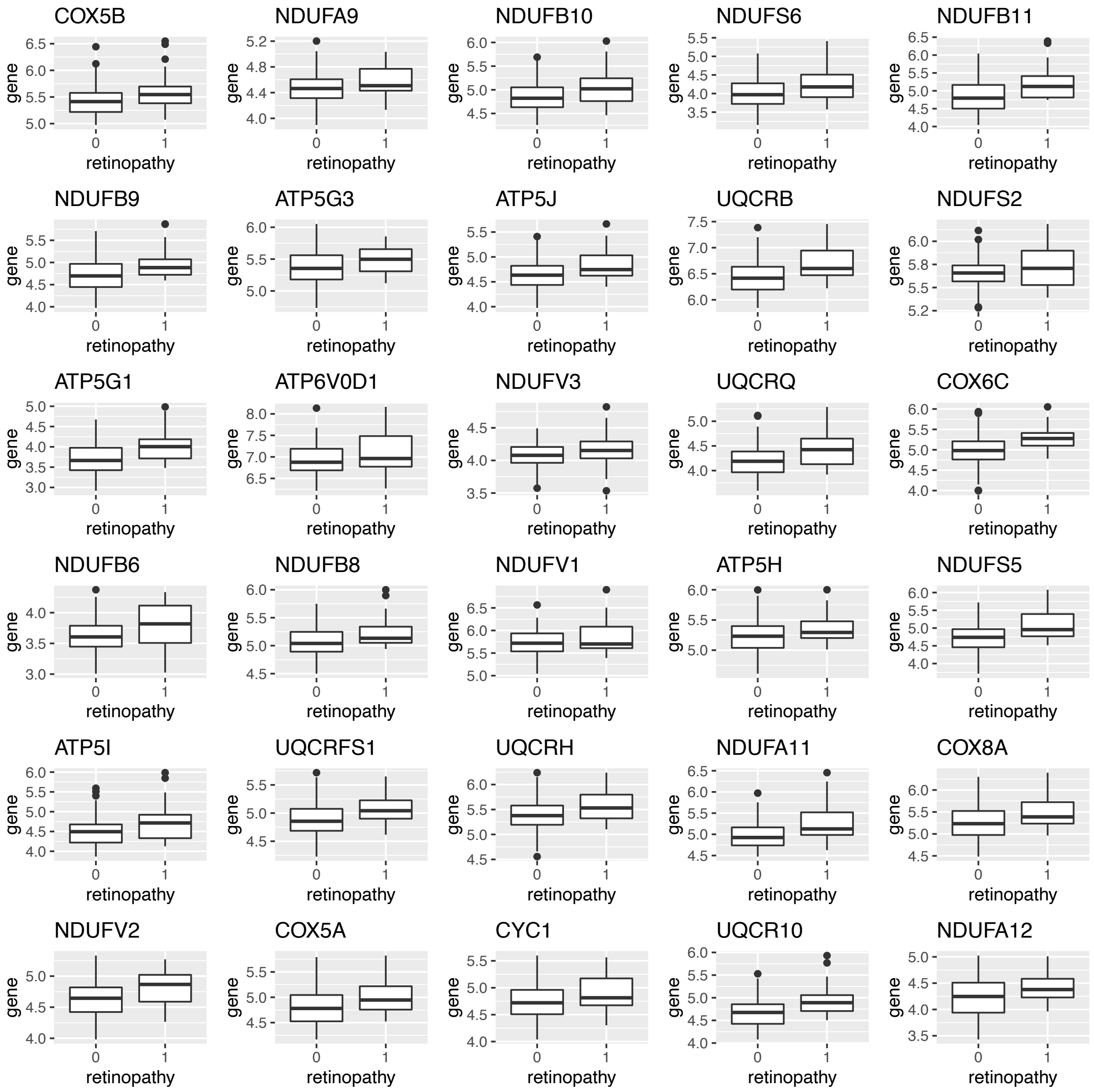

Figure S8b. OXPHOS gene expression changes between retinopathy/non- retinopathy for all genes in the pathway

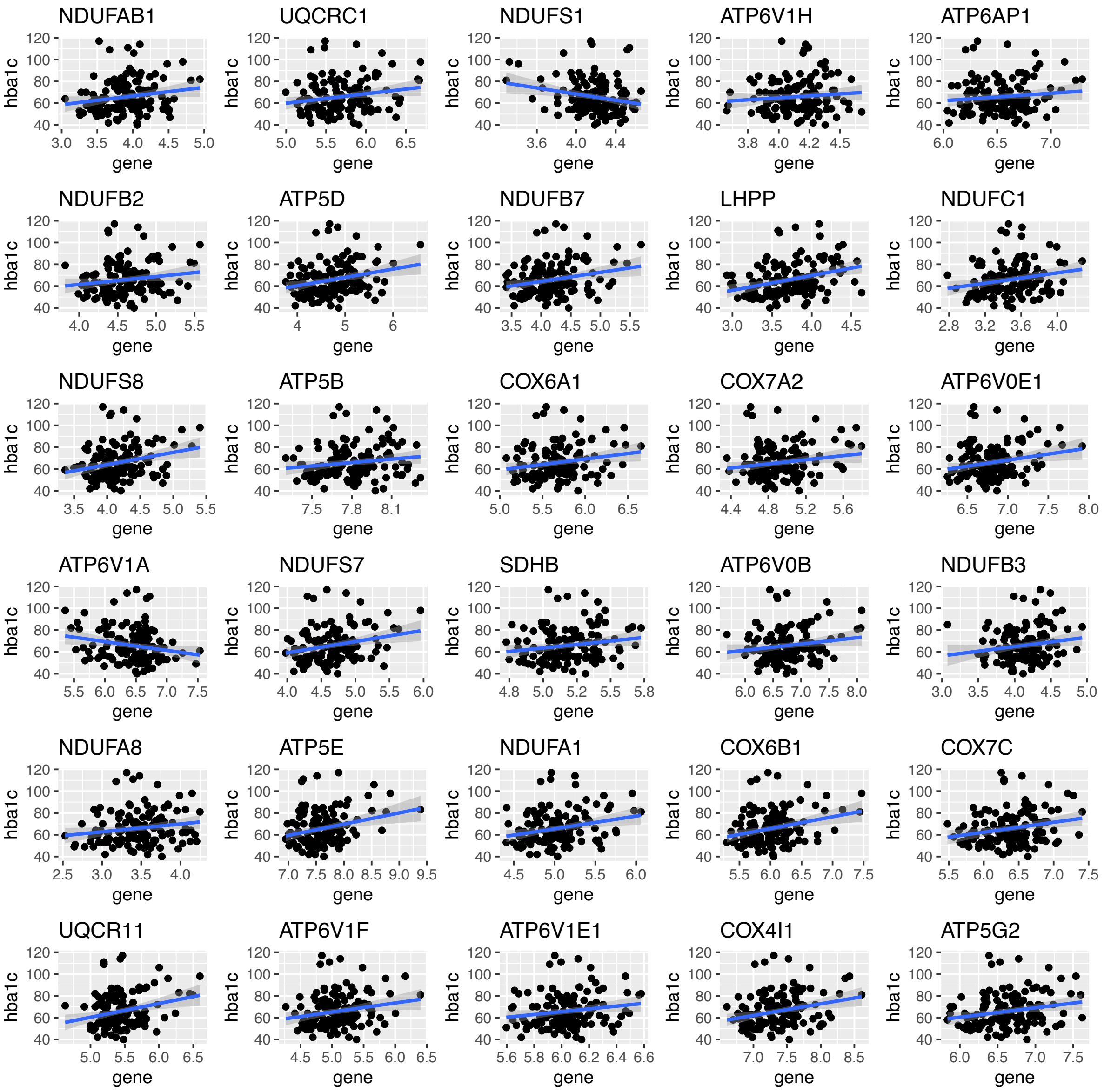

Figure S9a. OXPHOS gene expression versus HbA1c for all genes in the pathway

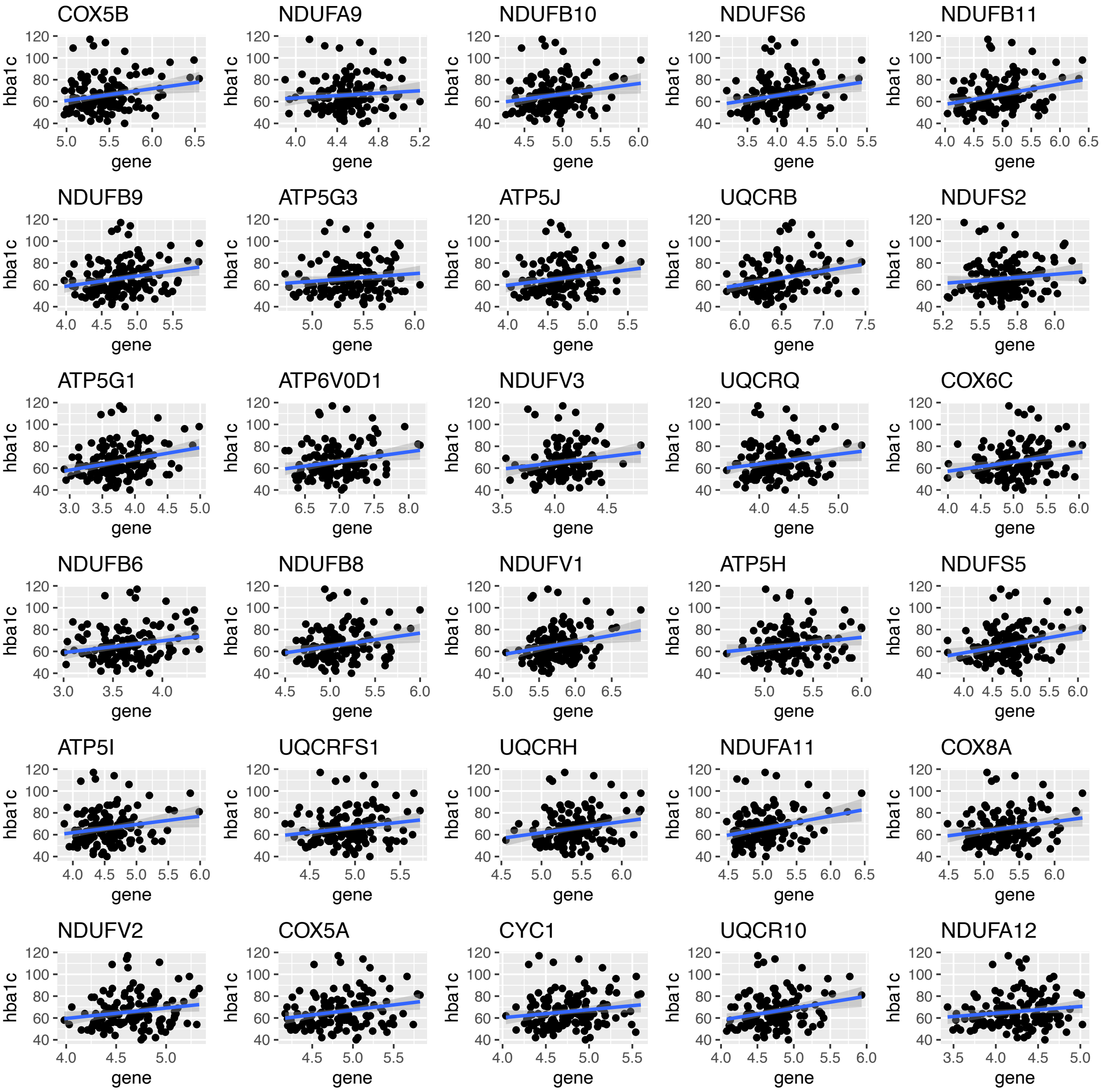

Figure S9b. OXPHOS gene expression versus HbA1c for all genes in the pathway

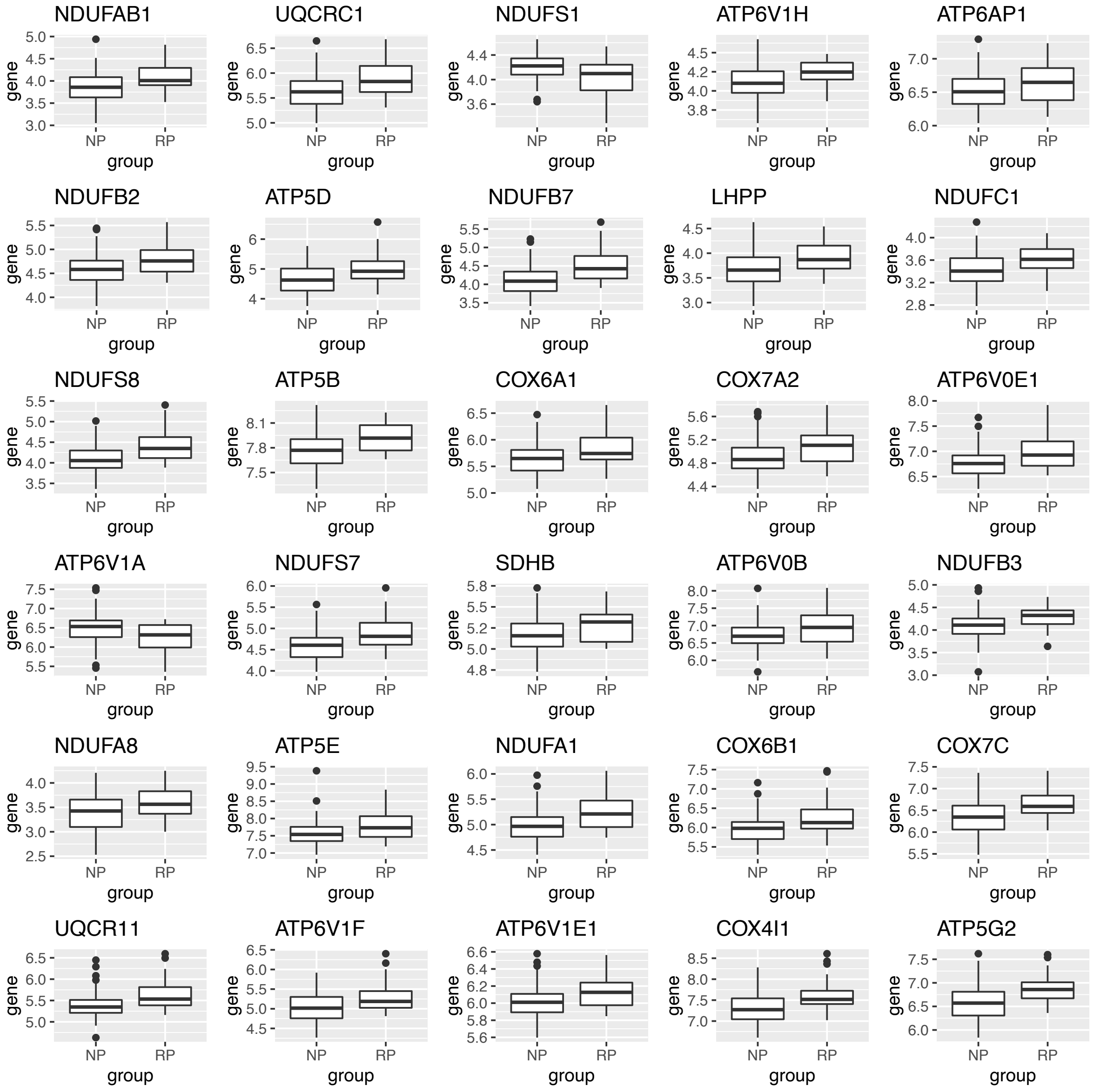

Figure S10a. OXPHOS gene expression changes between groups for all genes in the pathway

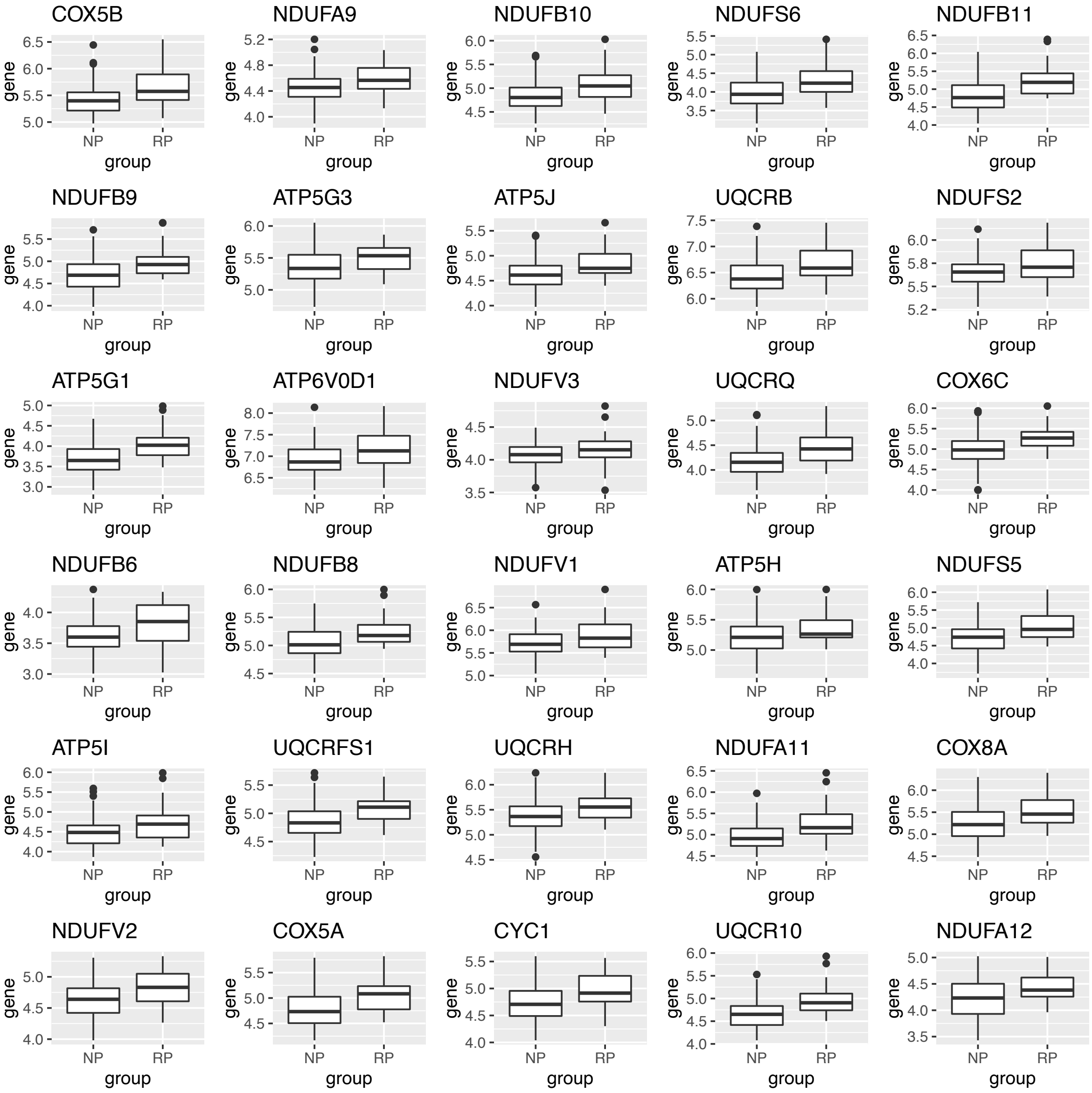

Figure S10b. OXPHOS gene expression changes between groups for all genes in the pathway

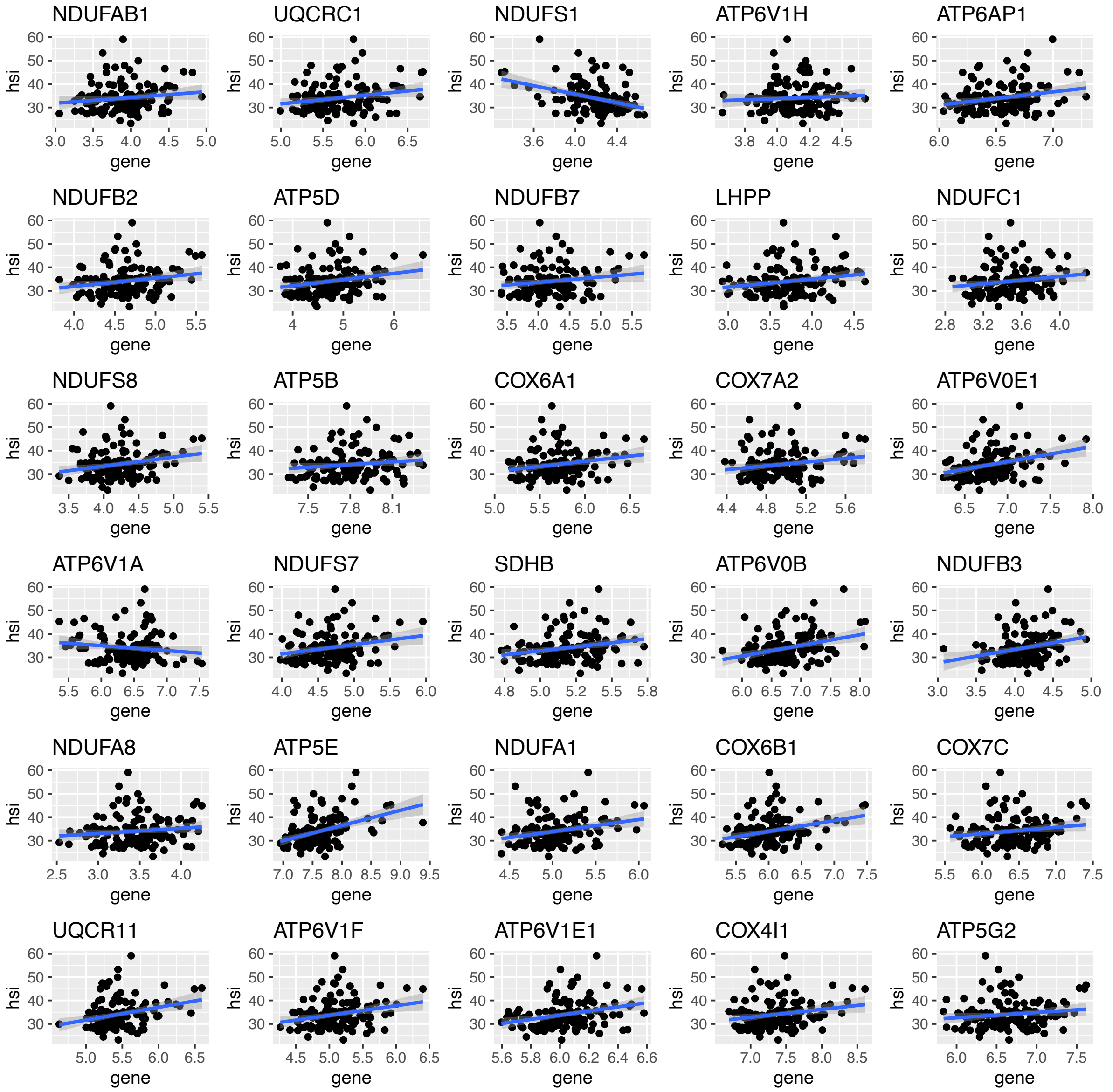

Figure S11a. OXPHOS gene expression versus HSI for all genes in the pathway

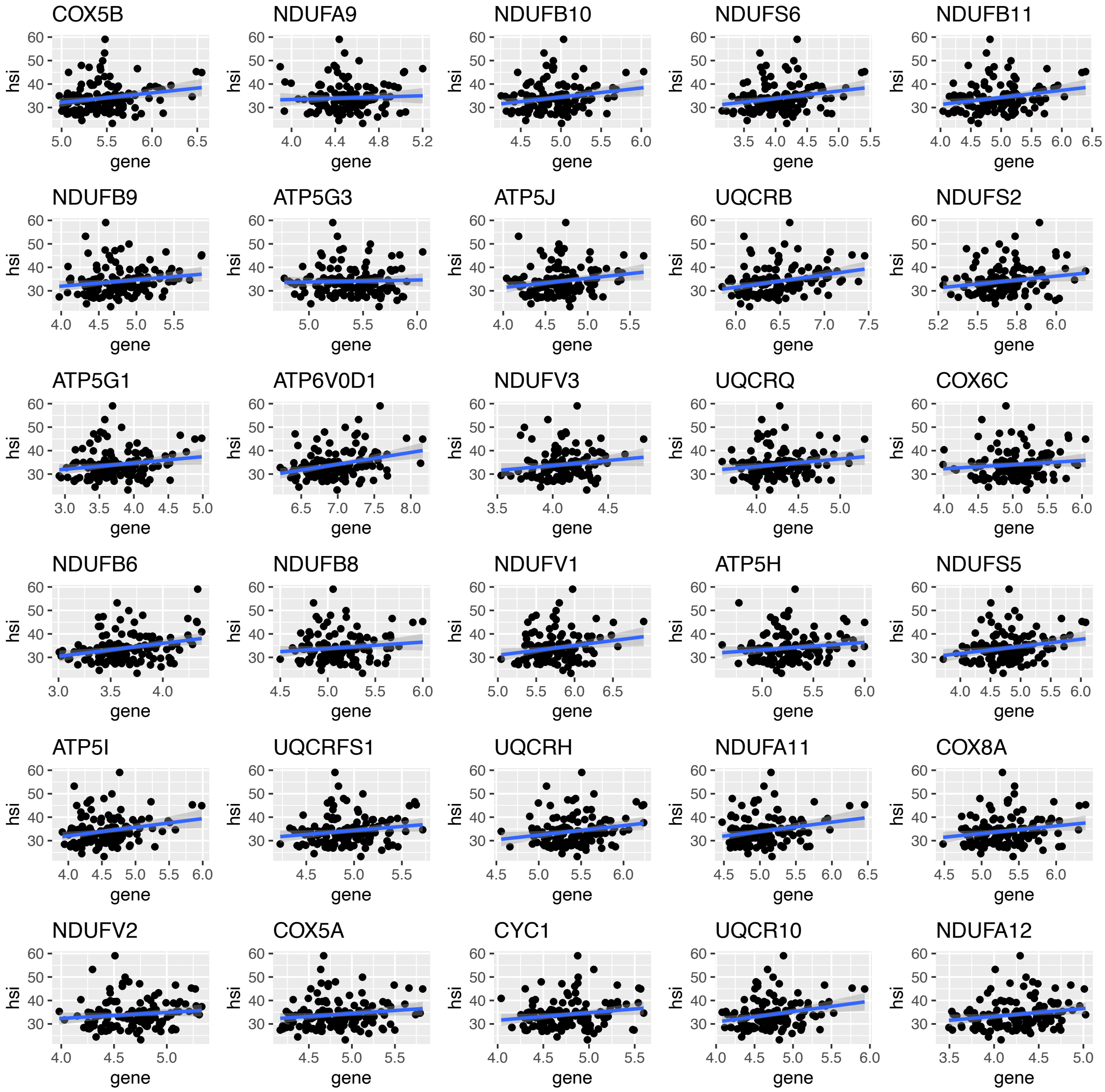

Figure S11b. OXPHOS gene expression versus HSI for all genes in the pathway

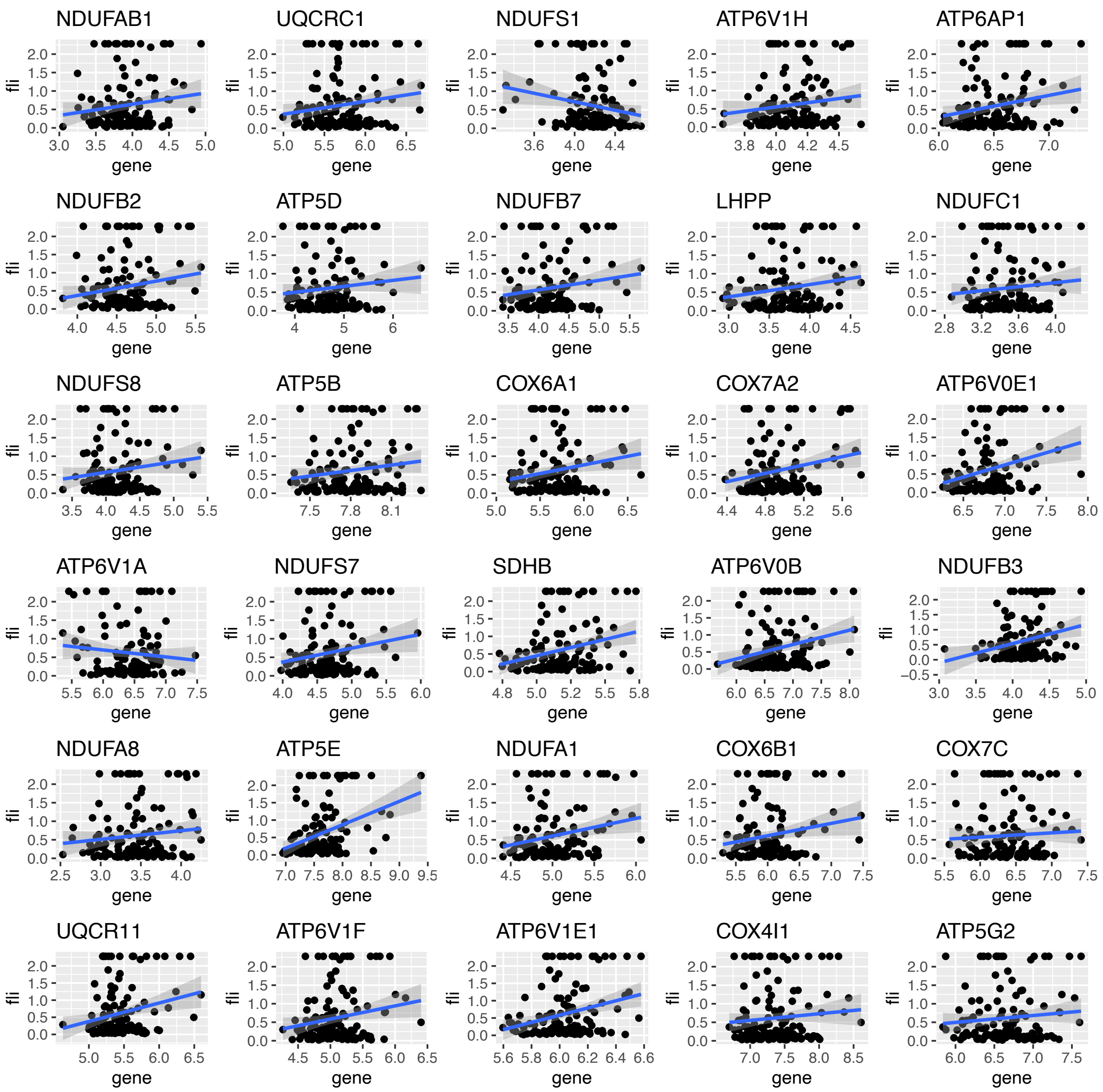

Figure S12a. OXPHOS gene expression versus FLI for all genes in the pathway

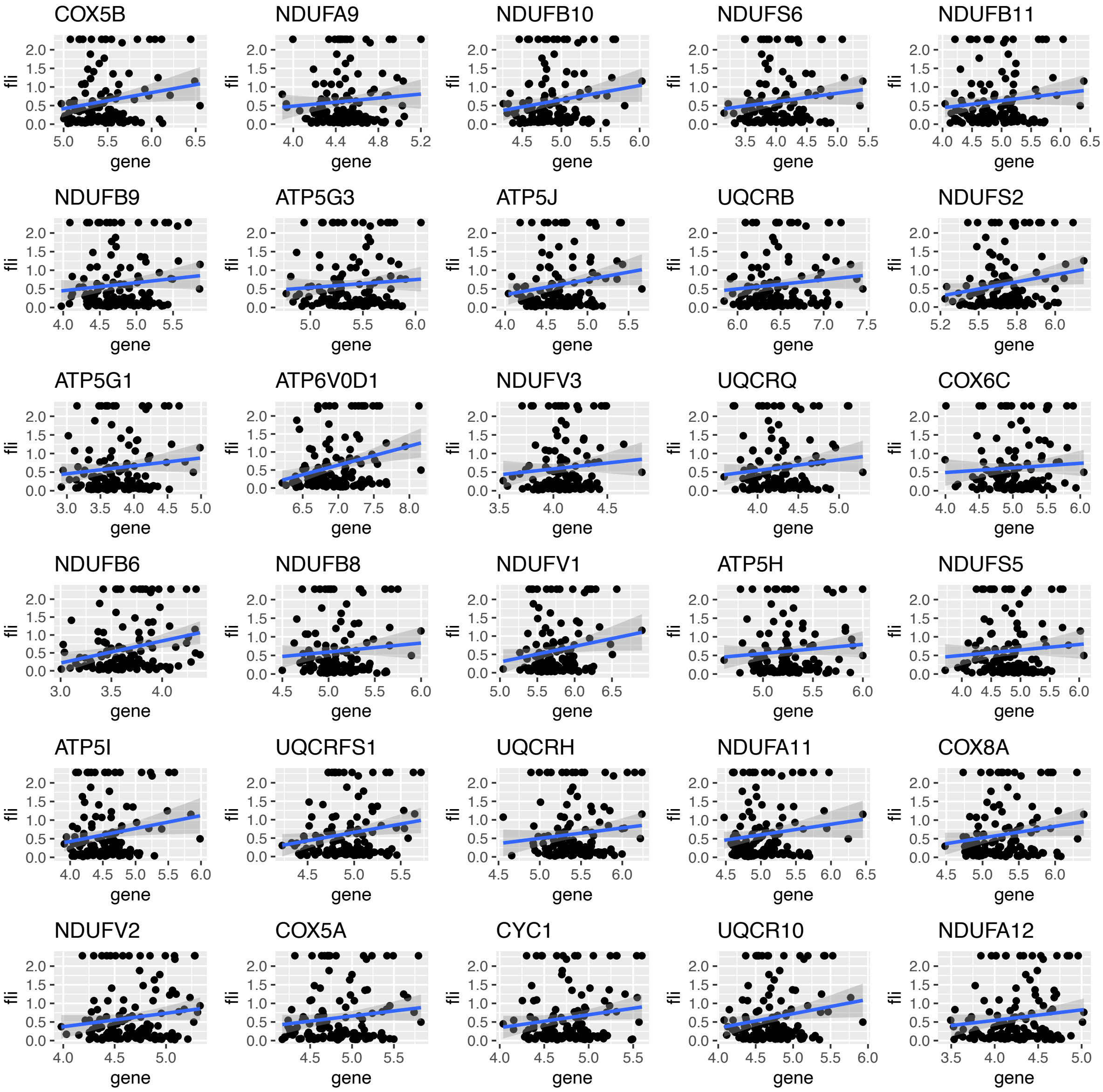

Figure S12b. OXPHOS gene expression versus FLI for all genes in the pathway

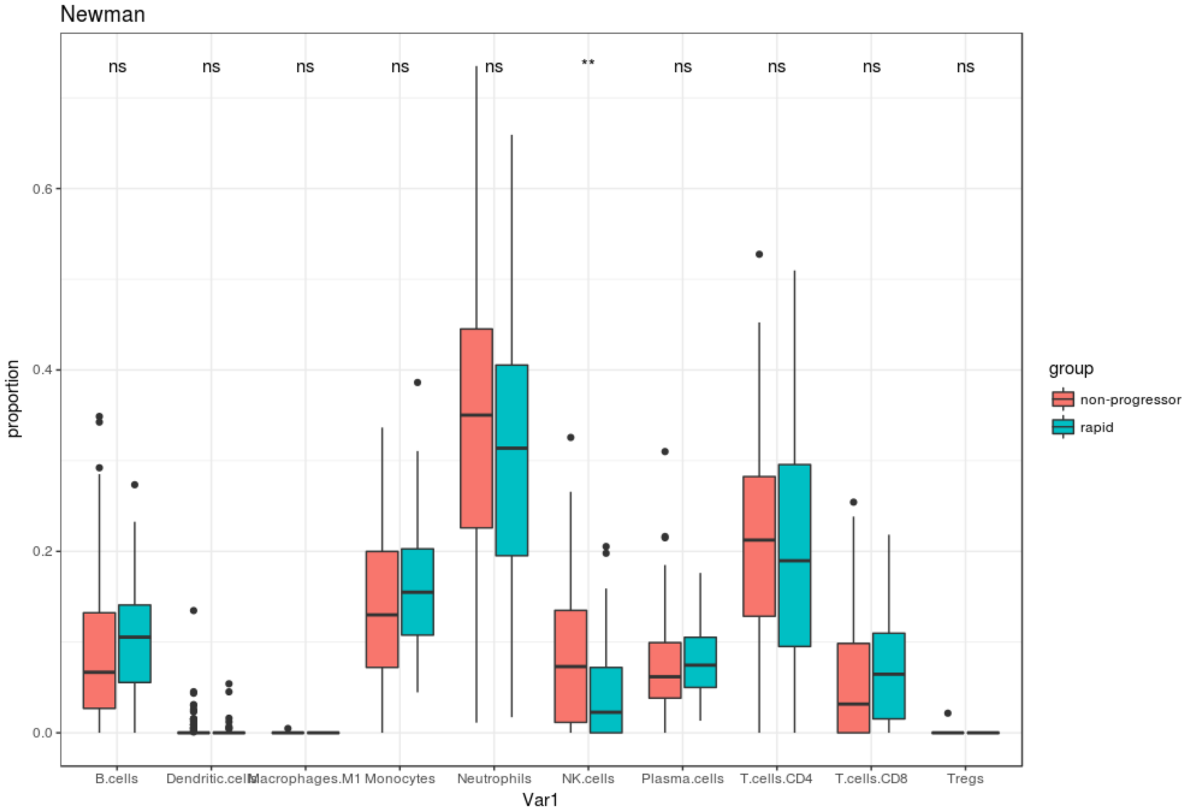

Figure S13. Cell deconvolution comparison between two groups using DeconRNASeq with using expression signature from ref. (44)

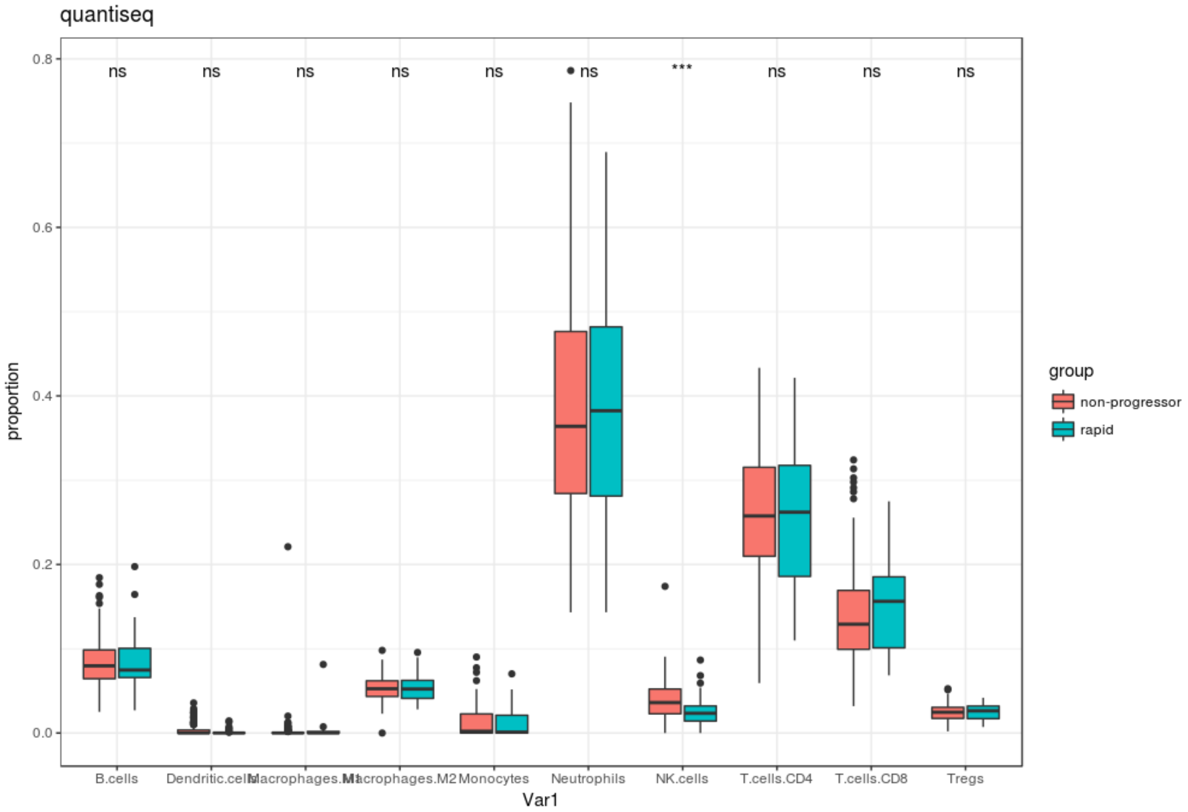

Figure S14. Cell deconvolution comparison between two groups using quanTiseq
